# Carbon Nanodots-Based Polymer Nanocomposite: A Potential Drug Delivery Armament of Phytopharmaceuticals

**DOI:** 10.3390/polym17030365

**Published:** 2025-01-29

**Authors:** Rabin Debnath, Abu Md Ashif Ikbal, Neeraj Kr. Ravi, Hanieh Kargarzadeh, Partha Palit, Sabu Thomas

**Affiliations:** 1 Department of Pharmaceutical Sciences, Drug Discovery Research Laboratory, Assam University, Silchar 788011, India; rabindnath26@gmail.com (R.D.); abu.md.ashif.ikbal@aus.ac.in (A.M.A.I.); neerajrav2095@gmail.com (N.K.R.); 2 Center of Molecular and Macromolecular Studies, Polish Academy of Sciences, Sienkiewicza 112, 90-363 Lodz, Poland; hanieh.kargar@gmail.com; 3 School of Energy Materials, School of Nanoscience and Nanotechnology, School of Polymer Science and Technology, School of Chemical Science and International, Inter University Centre for Nanoscience and Nantechnology (IIUCNN), Mahatma Gandhi University, Kottayam 686560, India; 4 Department of Chemical Sciences, University of Johannesburg, Doornfontein, Johannesburg P.O. Box 17011, South Africa; 5 TrEST Research Park, TC-4/2322, GEM Building, Opposite College of Engineering Trivandrum, Kulathoor Rd., Sreekariyam, Trivandrum 695016, India

**Keywords:** carbon nanodots, phytomedicine, polymeric carbon nanodots, drug delivery, CNDs

## Abstract

Carbon nanodots (CNDs) have garnered significant attention as viable drug delivery vehicles in recent years, especially in the field of phytomedicine. Although there is much promise for therapeutic applications with phytomedicine, its effectiveness is frequently restricted by its low solubility, stability, and bioavailability. This paper offers a thorough synopsis of the developing field of phytomedicine drug delivery based on CND. It explores CND synthesis processes, surface functionalization strategies, and structural and optical characteristics. Additionally, the advantages and difficulties of phytomedicine are examined, with a focus on the contribution of drug delivery methods to the increased effectiveness of phytomedicine. The applications of CNDs in drug delivery are also included in the review, along with the mechanisms that underlie their improved drug delivery capabilities. Additionally, it looks at controlled-release methods, stability augmentation, and phytomedicine-loading tactics onto CNDs. The potential of polymeric carbon nanodots in drug delivery is also covered, along with difficulties and prospective directions going forward, such as resolving toxicity and biocompatibility issues. In summary, the present review highlights the encouraging contribution of CNDs to the field of drug delivery, specifically in enhancing the potential of phytomedicine for therapeutic purposes.

## 1. Introduction

### 1.1. Background

**Figure 1:**
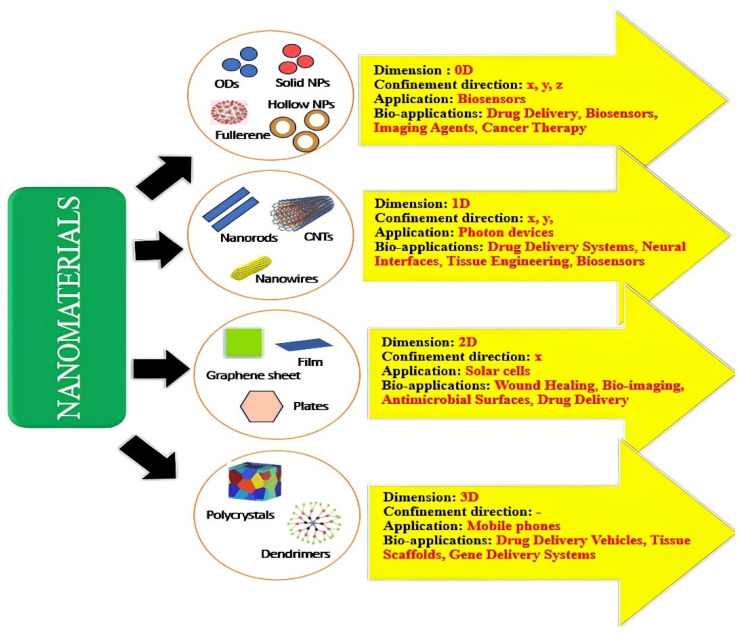
Different types of nanomaterials used in biomedical applications.

A class of nanomaterials known as carbon nanodots (CNDs) has caught the attention of scientists because of its special qualities and broad range of uses. The amazing features of CNDs, including their programmable surface chemistry, biocompatibility, and bio-adsorption, have led to increased interest in CNDs’ application in phytopharmaceutical drug delivery [1]. However, there are still flaws and shortcomings in the recent way that drugs are currently delivered for different dosage forms, such as problems with therapeutic efficacy, sustained release, and specificity. Carbon nanodots have attracted considerable attention owing to their distinctive advantages compared to other formulations. These nanodots possess unique properties, such as adjustable size, surface chemistry, and fluorescence characteristics, rendering them highly versatile for diverse applications. Additionally, their biocompatibility is noteworthy, with certain carbon nanodots demonstrating minimal toxicity, a vital feature for their potential biomedical use. Importantly, their synthesis is relatively straightforward, often achievable from readily available starting materials through simple methods. These attributes contribute to the growing interest in carbon nanodots across various fields, promising novel solutions for a wide range of applications. These tiny carbon-based particles usually exhibit a spherical or quasi-spherical morphology and range in size from 1 to 10 nanometers [2,3]. Carbon nanodots, which are made up of carbon atoms arranged in a sp^2^-hybridized structure similar to graphene, have attracted interest from a variety of scientific and technological fields [3]. Typical precursors comprise organic compounds such as glucose and citric acid, which offer a carbon-rich base for the creation of nanodots [4]. The size, surface chemistry, and the optical properties of the resultant carbon nanodots are all significantly influenced by the production process. The extraordinary optical qualities of carbon nanodots are one of their most notable characteristics. They are especially well-suited for use in bioimaging and noninvasive medical diagnostics because of their high fluorescence [5]. Their biocompatibility and imaging abilities suggest that they may be useful for targeted medication delivery and non-invasive diagnostics. Carbon nanodots have a high surface area and a distinct electrical structure that make them attractive candidates for a variety of uses [1]. Their surface is easily functionalized with various chemical groups, increasing their adaptability to many applications. Carbon nanodots have proven effective in sensing applications for a variety of analytes, such as metal ions, pH variations, and biomolecules. Surface functionalization is a simple process that enables customized interactions with particular target molecules, making them a useful platform tool in the production of sophisticated sensors for healthcare, environmental monitoring, and other industries [6]. Another application where carbon nanodots hold promise is catalysis. CNDs are effective catalysts for a variety of chemical processes due to their large surface area and distinctive electrical characteristics. Their catalytic potential is being investigated by researchers in a variety of processes, including environmental remediation and conventional chemical synthesis [7]. Carbon nanodots’ versatility in catalysis highlights the contribution they provide to the advancement of effective and sustainable chemical transformations. Carbon nanodots are used in energy-related sectors in addition to sensing and catalysis. They are intriguing possibilities for usage in solar cells and supercapacitors due to their distinct electrical characteristics [8]. Researchers are examining strategies that exploit the capabilities of CNDs to optimize energy storage devices’ efficiency and boost solar cells’ performance. Carbon nanoparticles’ biocompatibility and adjustable characteristics make them attractive for use in a variety of cutting-edge technologies [9]. A crucial feature of carbon nanodots that increases their potential for biological and medicinal applications is their biocompatibility. Because of their low toxicity, CNDs can be used in drug delivery systems to deliver therapeutic molecules to precise sites in a regulated way [10]. Their capacity to functionalize their surface with targeted ligands improves their precision in drug administration and presents a viable way to raise therapeutic treatment efficacy while lowering negative effects. The goals of carbon nanodot research continue to be improving synthesis techniques, finding new uses, and maximizing the qualities of the material for particular purposes. Current studies seek to learn more about the basic properties of CNDs, which will open the door to advancements in a variety of industries, including energy storage, sensing technologies, medicine, and catalysis. Carbon nanodot research is interdisciplinary, which highlights how revolutionary they can be for a variety of industries and how they can help create new kinds of innovative materials with never-before-seen capabilities [11]. Below, Figure 1 offers the different types of nanomaterials used for drug delivery.

### 1.2. Significance of CNDs in Drug Delivery Systems

In contemporary medicine, drug delivery systems are essential because they provide creative ways to improve the efficacy and security of therapeutic interventions. The importance of drug delivery systems resides in their capacity to resolve several issues related to traditional drug administration, which eventually enhances treatment outcomes and patient experiences [12]. These technologies seek to improve drug bioavailability, target distribution to particular body locations, and optimize drug pharmacokinetics. Achieving the best possible drug concentration at the intended location with the least amount of systemic exposure is a major difficulty in traditional drug delivery. Drug delivery systems make the controlled release possible, guaranteeing a consistent and therapeutically effective drug concentration across the intended time [13]. By doing so, drug level variations are avoided, adverse effects are decreased, and patient compliance is increased. Drugs that are poorly soluble, for instance, can be encapsulated by liposomes and nanoparticles to prevent degradation and enhance absorption. By ensuring that a higher percentage of the supplied medicine reaches the target, this increase in bioavailability maximizes therapeutic effects [14]. This is especially important for treating diseases like cancer when localized therapy is preferred. For example, passive or active targeting methods can be used to create nanoparticles and liposomes so that they preferentially aggregate in tumor tissues, enhancing drug delivery to cancer cells while sparing healthy tissues. For instance, the blood–brain barrier prevents many medications from entering the central nervous system. Bypassing or penetrating these barriers with drug delivery devices creates new opportunities for treating diseases affecting the brain and other difficult anatomical regions [15]. Drug delivery systems are important for getting beyond the body’s physiological barriers. Drug delivery systems are essential to the medical revolution because they improve the accuracy, effectiveness, and safety of therapeutic interventions. Because of its special qualities and biocompatibility, carbon nanodots (CNDs) have become one of the many nanomaterials being investigated for drug delivery [16]. Carbon nanodots’ small size promotes interactions at the cellular and molecular levels, so medicines can be delivered to particular tissues or cells with accuracy and efficiency. One important aspect of CNDs’ importance for drug administration is their biocompatibility [17]. Developing drug carriers that can successfully navigate the intricate biological environment and deliver therapeutic payloads without causing detrimental side effects requires biocompatibility [18]. The fluorescence in drug delivery systems primarily stems from their optical properties and creates opportunities for theragnostic applications, which combine therapy and diagnostics on a single platform, in addition to helping with bioimaging for real-time drug carrier tracking. Approaches to personalized medicine are made possible by the real-time visualization and monitoring of drug delivery mechanisms [19]. Despite limitations in emission of CNDs, which are usually absorbed by the tissues, Lee et al. demonstrated tissue imaging using nitrogen-doped carbon nanodots using photo acoustic imaging in the near IR region with high resolution. Authors even compared the response of nitrogen-doped CNDs with Gold Nanorods and Microbubbles which are used as contrast agents for photoacoustic imaging. CNDs showed higher heat conversion efficiency and stable photoacoustic signals than the other two contrast agents [20]. Carbon nanodots must be surface functionalized to be customized for a specific drug delivery application. Targeting ligands, medicinal compounds, or other biomolecules can be attached to CNDs by adding different functional groups on their surface; for example, carboxyl groups can be used to link anticancer drugs, and hydroxyl groups can facilitate the binding of DNA or RNA molecules for gene therapy [21]. Moreover, functionalized carbon nanodots can be designed to react to external factors like pH variations or certain enzymes, causing regulated drug release at the intended location. Carbon nanodots are used in drug delivery systems because of their ability to overcome biological barriers [22]. The central nervous system is protected from potentially dangerous substances by the blood–brain barrier (BBB). It is the highly discriminatory semipermeable border of endothelial cells that controls the transfer of solutes and chemicals between the central nervous system and the circulatory system. This protective function simultaneously poses a challenge to the delivery of therapeutic substances to the brain. Due of their small size and adaptable surface characteristics, carbon nanodots (CNDs) can be engineered to pass through the blood–brain barrier (BBB), opening up new therapeutic options for neurological disorders that are otherwise challenging to treat with traditional drug delivery techniques [23]. Additionally, there is plenty of room for loading and transporting therapeutic payloads on carbon nanodots due to their large surface area. CNDs offer a variety of drug-loading techniques, including encapsulation, adsorbing, and covalent attachment. In order to ensure that a sufficient amount of the therapeutic substance reaches the target site to exercise its intended effects, this loading capacity is crucial for optimizing drug delivery systems [24]. Beyond conventional small-molecule medications, carbon nanodots are incredibly versatile. A vast array of medicinal substances, including proteins, peptides, and nucleic acids (such as DNA and RNA), have been demonstrated to be delivered by them via their promise [25]. A major development in the realm of nucleic acid therapies and biological products is the capacity of CNDs to precisely deliver and protect sensitive biomolecules. To sum up, the importance of carbon nanodot-based drug delivery systems originates from their capacity to overcome the obstacles related to traditional drug administration [26]. CNDs are potential vehicles for targeted and regulated drug administration because of their special mix of characteristics: small size, biocompatibility, optical characteristics, surface functionalization, and barrier-crossing abilities. This field’s continued study and development has the potential to completely transform medical care by providing more individualized and effective therapeutic interventions with fewer adverse effects and better patient outcomes. The use of carbon nanodots in drug delivery systems offers optimism for the advancement of medicine towards safer, more accurate, and effective treatment modalities as our understanding of them improves [27].

### 1.3. Emergence of Carbon Nanodots in Drug Delivery

The scientific and medical communities are particularly interested in the use of carbon nanodots (CNDs) in drug administration because they present a fresh and exciting opportunity to improve drug delivery methods [28]. These nanodots are excellent prospects for a variety of biomedical applications, especially in the field of drug administration, due to their unique qualities, which include biocompatibility, biodegradability, eco-friendly, green-synthesis techniques, fluorescence, and a high surface area [29]. Due to their low toxicity and ability to be produced from organic precursors like glucose or citric acid, these nanodots rarely cause harm to biological systems. Their incorporation into medication delivery systems, where materials must effortlessly interact with the complex biological environment without causing harm, depends on their biocompatibility [30]. However, the CND to be used in the clinical settings must undergo rigorous toxicity studies as the toxicity of a CND depends not only on its surface modifications; it also depends on the surface charge and the role of functional group restricted by the said charge. For a CND to be biocompatible and non-toxic, its core must not ionize into a toxic species and lead to in vivo toxicity despite no evidence of toxicity in vitro. Neutral or negatively charged CNDs are found to be less toxic, whereas positively charged CNDs are most cytotoxic by initializing oxidative stress. However, its natural compatibility strongly depends on the precursor and on the production method used. Various kinds of surface functional groups can be obtained by inducing different responses and effects from the biological environment [31]. The fluorescence creates opportunities for theragnostic applications, in addition to allowing for real-time drug carrier monitoring and imaging. Theragnostic is the process of combining therapy and diagnostics into one platform to provide individualized and focused successful medical care. The fluorescence of carbon nanodots makes drug transport processes easier to see, improving the accuracy and effectiveness of therapeutic actions [32]. Charges and BBB-based delivery refers to drug delivery strategies that utilize either electrostatic charges or interactions with the blood–brain barrier (BBB) for targeted delivery of therapeutic agents to specific sites, particularly the brain. Utilizing charges and the blood–brain barrier (BBB) for drug delivery offers significant advantages in targeting neurological disorders. Drugs can be precisely delivered to the brain by taking advantage of charge-based interactions, and doing so maximizes therapeutic efficacy and minimizes systemic side effects. Furthermore, BBB-based delivery techniques allow medicines to pass through the barrier, removing a significant obstacle in the treatment of disorders of the central nervous system [33]. Surface functionalization is an important factor in the adaptability of carbon nanodots in drug delivery. Different functional groups can be added to the surface of CNDs with ease, enabling the attachment of medicinal compounds, targeted ligands, or other biomolecules. Drug carriers with particular affinities to target cells or tissues can be designed with this customization, which enhances drug delivery selectivity and reduces off-target effects [28]. Moreover, functionalized carbon nanodots can be designed to react to outside stimuli like pH variations or certain enzymes, causing regulated drug release at the intended location. The ability to interact at the nanoscale provided by the small size of carbon nanodots makes medication administration more accurate and effective by enabling the distribution of therapeutic substances to target tissues. Due to their small size and specific surface characteristics, nanoparticles can enter anatomical regions that would otherwise be inaccessible for targeted medication delivery. In difficult situations such as neurodegenerative disorders, nanoparticles provide a viable avenue for therapeutic treatments by utilizing both active surface changes and passive mechanisms, such as size and the Enhanced Permeability and Retention (EPR) effect [30]. This capacity is especially important for treating neurological illnesses, as traditional drug delivery techniques might not be able to get past these obstacles. Therapeutic payloads can be loaded and transported with plenty of room due to the high surface area of carbon nanodots. Different approaches for drug loading are available depending on whether the drug is covalently bonded, adsorbed, or encapsulated on the surface of CNDs. To maximize drug delivery systems and make sure that a sufficient amount of the therapeutic agent reaches the target site to have the desired effects, this loading capacity is crucial. Moreover, carbon nanodots’ growing importance in drug delivery is attributed to their ability to transport a diverse array of therapeutic molecules [34]. CNDs have demonstrated potential in delivering nucleic acids (such as DNA and RNA), proteins, and peptides in addition to conventional small-molecule medicines. This potential creates novel therapy options for cancer, genetic abnormalities, and other illnesses where the effectiveness of traditional medication delivery techniques may be constrained [25]. A major development in the realm of nucleic acid therapies and biologics is the capacity of CNDs to precisely deliver and protect sensitive biomolecules. To sum up, the application of carbon nanodots in drug delivery is a revolutionary step forward for the field of nanomedicine. Their nanoscale size, surface functionalization abilities, optical characteristics, and biocompatibility make them useful and efficient carriers of medicinal drugs. Carbon nanodots have the potential to completely transform drug delivery systems as long as an investigation into this field is conducted. They can provide novel approaches for the precise, targeted, and controlled delivery of medicines of various medical applications [35] in both life-threatening and chronic illnesses, including cancer, viral diseases, neurodegenerative disorders, etc.

## 2. Phytomedicine in Drug Delivery

A timeless field with roots in ancient wisdom, phytomedicine is embracing the cutting edge of modern drug delivery technologies while navigating the prevailing modern currents [36]. The combination of current novel drug delivery technology and phytomedicine may explore the Carbon Nanodots-Based Drug Delivery of Phytomedicine, lies at the crossroads of history and innovation, opening the door to therapeutic techniques that go beyond conventional practices [37]. The range of options available to us in phytomedicine becomes clear as we explore further. Beneath the abundance of bioactive substances is a dynamic interplay of holistic healing, an idea engrained in conventional medical practice. With a focus on overall health, phytomedicine aims to treat illnesses’ underlying causes, as well as their symptoms [38]. The symbiotic link between sophisticated medication delivery technologies and phytomedicine is a prominent feature of the modern period [39]. Although the ancient healers had an innate understanding of the power of plant treatments, current technology allows us to fully realize their potential through targeted and deliberate delivery methods [40]. This combines the best aspects of conventional medicine with the cutting-edge techniques of contemporary medication delivery.

### 2.1. Carbon Nanodots (CNDs): Catalysts of Transformation

Carbon nanodots (CNDs), heralded as revolutionary agents in drug delivery, are fundamental to this metamorphosis. CNDs have their roots in nanotechnology and offer of several unique features. They are ideal phytomedicinal chemical carriers due to their large surface area, nanoscale particle size, and configurable surface functionalities. This increases the bar for phytomedicine since CNDs’ biocompatibility and flexibility allow for more focused, accurate, and efficient drug administration [41].

#### 2.1.1. Precision Delivery for Optimal Impact

The use of CNDs in medication delivery systems is going to revolutionize precision medicine. Targeting the delivery of phytomedicine to specific tissues or cells becomes possible. The utilization of ancient plant knowledge in conjunction with nanoscale precision maximizes therapeutic effects while mitigating adverse consequences [42]. Theoretical capabilities of traditional healers become tangible with the tailored delivery mechanisms made possible by CNDs [43,44]. The sophistication that may be gained when innovation and tradition come together is demonstrated by this exact delivery method.

#### 2.1.2. Addressing Bioavailability Challenges

Though widely recognized for its comprehensive healing properties, phytomedicine encounters absorption challenges coupled with pharmacokinetic failure that could potentially diminish its therapeutic impact [45]. In this situation, CNDs are crucial for overcoming these barriers. Due to its ability to conjugate or encapsulate phytomedicinal compounds in nanodot flatform, it ensures that these bioactive substances are more soluble and stable and reach their target in a form that body can easily absorb [46]. The synergistic combination of phytomedicine and CNDs provides a modern answer to the historical problem of realizing the full potential of plant-derived therapies. CNDs are sentinels that protect stability and prolong the duration of therapeutic efficacy. CNDs provide protection against degradation, which has the potential to diminish the effectiveness of phytomedicines over time. These nanoscale entities stabilize the plant and guarantee that its medicinal properties hold true over time [47]. The resulting synergy bears witness to phytomedicine’s adaptable character, which welcomes innovation while maintaining its core principles. The voyage transcends the traditional and modern dichotomies as we navigate this complex confluence, creating a story where the best practices from each era join together to provide the best possible therapeutic results. The investigation of “Carbon Nanodots-Based Drug Delivery of Phytomedicine” turns into a journey not just through the pages of history and the halls of contemporary science, but also towards a future in which the harmonious fusion of traditional knowledge and cutting-edge technology shapes the face of healthcare.

#### 2.1.3. Guardians of Therapeutic Efficacy

Stability is essential for any drug delivery system to work, and in this regard, CNDs act as guardians. By stopping the degradation of phytomedicinal compounds, CNDs extend the duration of therapeutic activity. Although this property is not observed for every phytomedicine-derived CNDs, CND formulation of phytomedicine. This was observed in case of *Asparagus racemosus* [48]. This property is not observed in case of every CND derived from phytomedicine. But still CNDs act as a guardian of therapeutic efficacy. Despite absence of all anti-cancer active components in ginger–CDs except curcumin, they executed anti-cancer activity when prepared via hydrothermal method. Furthermore, Artemisiae Argyi Folium (AAF) was not indicated traditionally for anti-frostbite effects, still the CDs showed anti-frostbite activity by improving local inflammation and reducing blood glucose levels caused by frost bite despite the absence of isochlorogenic acid which is the major composition of AAF [49]. This stabilization ensures that phytomedicine’s therapeutic potential persists throughout time, offering a dependable and durable solution for a variety of medical needs. In general, a high temperature-induced hydrothermal or calcination process degrades thermos-degradable flavonoids, terpenoids, and polyphenols to synthesize macromolecular carbon polymeric composites. The functional group present in the alkaloids, terpenoids, and flavonoids can functionalize the surface of polymeric carbon nanodots to offer significant and improved biological response.

Therefore, it is clear from this combination of phytomedicine and enhanced drug delivery, especially when seen through the lens of carbon nanodots-based drug delivery, that this is both a scientific study and a reflection of how medicine is evolving. This symbiotic partnership, with its traditional foundation and new drive, has the power to fundamentally alter the way we view healing. As we traverse this complicated intersection of conventional treatments and cutting-edge technologies, we get a glimpse of a future in which the best features of both worlds come together to give the best possible therapeutic outcomes.

### 2.2. Overview of Phytomedicine

In order to properly contextualize the intricate tale of “Carbon Nanodots-Based Drug Delivery of Phytomedicine”, it is imperative that we first discuss the historical background and progression of phytomedicine. This overview serves as a bridge between the State-of-the-Art findings in the ground-breaking field of carbon nanodots (CNDs) and the conventional wisdom of plant-based therapy.

#### 2.2.1. Historical Roots and Evolution

Throughout the ages of human civilization, phytomedicine has left enduring cultural imprints. Because they possessed an instinctive understanding of nature’s pharmacy, healers from ancient times have exploited the medicinal properties of plants for millennia [50]. From Native American herbalists to Indian Ayurvedic sages, the lengthy history of phytomedicine attests to its ongoing efficacy. But as we learn more about “Carbon Nanodots-Based Drug Delivery of Phytomedicine”, the historical continuum becomes more meaningful. It becomes proof of how flexible phytomedicine is, fitting in seamlessly with the evolving landscape of medical practices.

#### 2.2.2. Bioactive Compounds in Phytomedicine

Examining the enormous pool of bioactive compounds found in phytomedicine reveals an unparalleled diversity in plant pharmacopoeia. Alkaloids, flavonoids, terpenoids, and polyphenols come together to provide a complex mosaic of therapeutic effects [51]. This symphony of bioactive molecules, with origins in conventional medicine, paves the way for the inclusion of carbon nanodots. Every class of chemicals engages in a cooperative interplay within the plant matrix, in addition to possessing distinct therapeutic characteristics. The originality of CNDs is based on the synergy that is the hallmark of phytomedicine.

As we explore the complex environment, it becomes clear that the bioactive substances in phytomedicine are not merely stand-alone treatments but rather integral parts of a whole healing process. Moreover, the interdependence within the plant matrix becomes a driving force behind the integration of contemporary medication delivery systems.

### 2.3. Merits and Demerits of Phytomedicines

#### 2.3.1. Advantages of Phytomedicine

Phytomedicine is a medicinal method with several benefits that is based on the use of chemicals produced from plants [52]. These therapeutic substances have a strong cultural and traditional legitimacy due to their natural origin and long history of use. Plants include a wide range of bioactive chemicals, such as alkaloids, flavonoids, terpenoids, and polyphenols, which together contribute to their diverse and abundant medicinal capabilities [53]. This diversity highlights the holistic character of phytomedicine by enabling the treatment of various illnesses with a single plant or extract. Furthermore, in keeping with the tenets of personalized medicine, the holistic healing approach goes beyond treating symptoms to address the root causes of illnesses. When it comes to side effects and safety, phytomedicine frequently has an advantage over synthetic medications. The complex interplay between the many chemicals found in plants frequently reduces the negative effects of their isolated synthetic counterparts. Moreover, phytomedicine is an affordable and easily accessible alternative to traditional medicine in areas where healthcare practices are strongly rooted in traditional medicine [54]. Phytoextracts are a rich source of natural substances that can be used as carbon sources for nanoparticle synthesis, which is why they are used as precursors for the development of Carbon Nanodots (CNDs). Many organic components found in phytoextracts, including proteins, phenolic compounds, and carbohydrates, can be carbonized to produce polymeric carbon-based nanoparticles like CNDs, using a hydrothermal calcination process. This strategy provides an environmentally benign and sustainable way to synthesize

#### 2.3.2. Challenges of Phytomedicine

Despite all of its benefits, phytomedicine faces a number of obstacles that require careful thought [55]. Among these difficulties, standardization and quality control rank first. The natural fluctuation in plant growth environments, harvesting schedules, and extraction techniques makes it more difficult to create constant potency and effectiveness. Strict protocols are therefore required to guarantee consistency throughout various phytomedicine batches. One of the main obstacles to the widespread application of phytomedicines in conventional medicine is the lack of knowledge regarding their complex mechanisms of action [56]. To properly understand these systems and incorporate them into traditional healthcare methods, more research is necessary. Another obstacle to the efficient application of phytomedicines is their bioavailability. When taken orally, many bioactive chemicals have low bioavailability, which restricts their potential applications as therapeutic agents. Developing novel strategies to improve these chemicals’ availability and absorption within the body is necessary to meet this problem. Furthermore, much research is required to clarify and appropriately manage the possible interactions between phytomedicines and conventional pharmaceuticals [57]. Cultural prejudices and the various legal systems around the world could make it difficult for phytomedicine to become widely accepted, necessitating coordinated efforts to close these gaps.

### 2.4. Advantages of Carbon Nanodots-Based Drug Delivery for Phytomedicine

The incorporation of carbon nanodots into phytomedicine drug delivery systems represents a paradigm change, opening up new avenues for overcoming long-standing obstacles. There are numerous benefits, the most notable of which is the notable increase in bioavailability. Carbon nanodots have the capacity to enhance the solubility and stability of phytomedicines, thereby guaranteeing the best possible absorption and utilization within the body [58]. This discovery could greatly increase the medicinal benefits of substances obtained from plants. One of the most revolutionary benefits is that carbon nanodots can deliver drugs specifically to the right places. By means of careful engineering, these nanodots can be precisely engineered to overcome physiological barriers and target individual tissues or cells. By limiting off-target effects, this focused delivery maximizes therapeutic benefit and minimizes negative outcomes. By extending the release of phytomedicines over prolonged periods of time, carbon nanodots’ controlled-release properties facilitate therapeutic optimization even more. In addition to increasing efficacy, this regulated distribution encourages patient compliance, which is essential for positive therapeutic results. In addition, the combination of phytomedicines and carbon nanodots offers the exciting possibility of synergistic effects. The utilization of these nanoscale carriers in conjunction with the complex blends of bioactive compounds present in plants has the potential to yield enhanced therapeutic effects, perhaps outperforming traditional drug delivery methods [40]. CNDs’ surface functional groups, such as carboxyl, hydroxyl, and amino groups, render suitable anchoring with the bioreceptors in the target zone to elicit impressive pharmacological action. Positively charged CNDs can effectively electrostatically bind with negatively charged bacterial membranes to attenuate the microbial infection very rapidly [59]. This positively charged surface of CNDs also assists them to penetrate the BBB to interact with negatively charged beta-amyloid precipitated protein to attenuate the neurodegenerative disorders to a greater extent [60].

### 2.5. Challenges of Carbon Nanodots-Based Drug Delivery for Phytomedicine

Although carbon nanodots have several benefits in phytomedicine, integrating them is a difficult task. The most important of these is the absolute necessity of a thorough safety assessment. It is crucial to evaluate the possible toxicity and enduring impacts of carbon nanodots on human anatomy prior to their extensive clinical usage. Logistically difficult, the complicated synthesis of carbon nanodots demands careful attention to quality control and reproducibility when scaling up production for commercial application [61]. Clearance and biocompatibility concerns are important aspects that need careful examination. For safe and efficient use, it is essential to make sure that carbon nanodots blend in with the biological environment and to comprehend what happens to them inside the body. The complex regulatory environment around nanodot-based drug delivery devices calls for the creation of particular approval criteria [62]. Strict adherence to these recommendations is necessary to guarantee the moral and secure incorporation of this cutting-edge technology into standard medical procedures. Therefore, integrating carbon nanodots into phytomedicine drug delivery systems is a revolutionary step towards addressing long-standing obstacles. Although the benefits are great, resolving safety issues, producing nanodots on a large scale, and negotiating regulatory hurdles are essential stages in bringing this novel method to its full potential in clinical settings [63]. In order to guarantee the smooth integration of carbon nanodots with phytomedicine and launch a new era of precision medicine, a multidisciplinary and thorough approach is essential.

### 2.6. Role of Drug Delivery Systems in Enhancing Phytomedicine Efficacy

The medicinal potential of phytomedicine, which comes from plant sources, has been highly esteemed [64]. On the other hand, intrinsic difficulties such as low bioavailability, restricted targeting, and regulated release may compromise its effectiveness. One revolutionary way to address these issues and greatly improve the overall effectiveness of phytomedicine is the use of cutting-edge drug delivery systems, with a focus on those based on carbon nanodots [65].

#### 2.6.1. Enhanced Bioavailability

Low bioavailability is a common problem for phytomedicines, mostly because of things like poor solubility. Because of their distinct surface characteristics and nanoscale size, carbon nanodots are effective carriers to overcome this constraint. By increasing the solubility of hydrophobic phytomedicine components, they can optimize bioavailability by facilitating improved absorption and distribution within the body [66].

#### 2.6.2. Targeted Drug Delivery

The remarkable ability of carbon nanodots for targeted drug delivery is an important factor in optimizing treatment efficacy while reducing side effects. These nanodots’ functionalization and surface alterations enable exact customization to target particular tissues or cells [22,67]. By ensuring that phytomedicines reach their intended site of action, this focused method maximizes therapeutic benefits and reduces off-target effects. By linking targeting molecules, such as antibodies and receptor ligands, to carbon nanodots help them locate specific cells in order to achieve targeted drugs delivery. Their surface is hydrophilic, which helps them attach to biological tissues in the body and avoid immune system clearance. By ensuring that the drug is released precisely where it is needed, innovative compounds that respond to pH changes can improve treatment and minimize adverse effects. Phytomolecule-derived pegylated CNDs can stabilize much better than empty CNDs without phytocompounds. Moreover, very low-sized carbon nanodots ranging from 10 to 30 nm, as evident from the literature, easily can uptake by the reticular endothelial cellular system without breakage [19] or damage for better efficacy including predicted delivery in low doses and frequency [68].

#### 2.6.3. Controlled Release

Attaining long-term therapeutic concentrations is essential for maximum effectiveness. One unique benefit of carbon nanodots is their ability to release phytomedicines under controlled conditions. Modulating the release kinetics guarantees a steady and progressive delivery, which not only improves efficacy but also encourages patient compliance by lowering the frequency of doses [58].

#### 2.6.4. Synergistic Effects

There is a chance that the combination of phytomedicines with carbon nanodots will have synergistic effects. Increased stability and activity may result from interactions between nanodots and the various bioactive substances found in phytomedicines [48]. When combined, these components have the potential to produce more potent therapeutic effects than those of single or conventional drug delivery methods.

#### 2.6.5. Improving Cellular Uptake

Carbon nanodots’ distinct surface characteristics and nanoscale size allow for improved cellular absorption of phytomedicines. This is especially important when it comes to intracellular targets. As effective carriers, the nanodots can help phytomedicines get through cell barriers and increase their total cellular bioavailability, which boosts their therapeutic effect. CNDs enhance cellular absorption of phytomedicines, potentially aiding uptake by macrophages and overcoming cellular barriers and could potentially aid in the uptake of phytomedicines by macrophages and other reticular endothelial system constituents [69].

Carbon nanodots (CNDs) have the potential to modulate and refine the immune system’s responses. They can provide therapeutic benefits in diseases involving immunological dysregulation, such as infections or autoimmune diseases, by regulating immune activity and stimulating it when needed to avoid overreactions. Notwithstanding these positive results, more investigation is necessary to completely understand the immunomodulatory effects of CNDs and maximize their use in clinical contexts. Gaining insight into the ways in which CNDs engage with the immune system may open up new options for treatment approaches and illness control techniques [70].

#### 2.6.6. Biocompatibility and Reduced Toxicity

Biocompatibility is essential for any medication delivery device to work. When thoughtfully developed, carbon nanodots exhibit low toxicity and great biocompatibility [71]. Their compatibility with biological systems reduces the possibility of negative consequences, and their biodegradability guarantees low long-term influence, all of which add to the overall safety of the phytomedicine delivery process. Specific solutions, such as surface modifications of carbon nanodots to reduce toxicity and enhance interaction with biological tissues helps in solving biocompatibility issues. Polyethylene glycol (PEG) is frequently applied to the surface of CNDs as a typical method of PEGylation, which reduces toxicity and increases stability in biological system. Moreover, CNDs can be coated with biopolymers, including hyaluronic acid or chitosan, to improve their uptake and decrease immunological responses by increasing their contact with cells. By functionalizing CNDs with targeting ligands, such as peptides or antibodies, they can bind to particular tissues or cells more specifically, minimizing off-target effects and enhancing biocompatibility [69]. The efficacy and safety of CNDs in drug delivery applications are greatly enhanced by these strategies.

#### 2.6.7. Personalized Medicine Approach

The adaptability of carbon nanodots makes personalized medicine possible, enabling the customization of drug delivery systems according to the unique characteristics of each patient [72]. By tailoring the phytomedicine to each patient’s unique needs and response, it might potentially minimize side effects and maximize therapeutic success.

To summarize, the application of drug delivery systems, especially those that make use of carbon nanodots goes beyond the traditional constraints connected to phytomedicine. Carbon nanodots present a novel platform for improving the effectiveness of phytomedicines by resolving issues with bioavailability, targeted distribution, controlled release, and promoting synergistic effects. In the field of natural therapies, this not only provides answers to current problems but also opens doors to a new era of precision and personalized therapy. It is highly promising that the integration of these cutting-edge drug delivery methods will transform the therapeutic landscape and enable phytomedicine to reach its full potential in clinical applications.

## 3. Carbon Nanodots: Properties and Synthesis

Carbon nanomaterials have been attracting a great deal of research interest in the past decades due to their unique properties such as biocompatibility, nontoxicity, high mechanical and thermal properties, easy functionalization, and possessing of photoluminescent nanomaterials. It also has strong absorption, high quantum yield, shows promising optical, energy, and biomedicine applications. Among all carbonaceous nanomaterials, carbon dots (CNDs), were first obtained during the purification of single-walled carbon nanotubes through preparative electrophoresis by Scrivens and co-workers in 2004 [73].

Over time, CNDs have been proven to have many excellent properties. Additionally, increasing interest has been observed in the utilization of CNDs in the fields of energy and catalysis [74,75], biological labelling, bioimaging, and gene/drug delivery [76,77]. This increased interest is attributed to their unique properties, such as water-solubility, low toxicity, high chemical stability, and easy functioning.

### 3.1. Sources, Structures, and Properties of Carbon Nanodots

Carbon-based substances like fruit, botanical extracts and citric acid are known as organic precursors in the synthesis of carbon nanodots (CNDs). On the other hand, inorganic precursors provide other elements that influence the properties of CNDs. The flexibility and environmental friendliness of CND synthesis techniques are demonstrated by the use of a variety of feedstock, including green carbon precursors like fruit peels and kitchen trash. Various chemical precursors have been identified for the synthesis of CNDs, including organic and inorganic precursors, such as ammonium citrate [78], ethylene glycol [79], citric acid [80], Ethylene diamine tetra acetic acid (EDTA) [81], phytic acid [82], phenylenediamine [83], thiourea [84], carbon nanotube [74,75], graphite [85], etc. Meanwhile, a large number of green carbon precursors have been used to produce CNDs, including fruits, fruit juices, and fruit peels [86,87,88]; animal and animal-derived materials, such as chicken eggs [89] and silkworm [90]; vegetables and spices [91], waste kitchen materials, like frying oil [92] or waste paper [93]; plant leaves and derivatives [94]; etc.

It is worth noting that, while CNDs typically showcase a dot-like structure in TEM, introducing unconventional precursors like rods, ribbons, and triangles can yield unexpected outcomes. Although this diversifies the appearance of CNDs, it concurrently heightens the challenge of uncontrollable preparation. Successful management of this process necessitates accurate prediction of CND formation and strategic selection of precursors [95].

CNDs are classified into three main groups, graphene quantum dots (GQDs), carbon nanodots (CNDs), and polymer dots (PDs) [96]. GQDs possess a crystalline structure and consist of a single or a few graphene layers with π conjugation. Chemically, it has more functional groups on the edges and shows an anisotropic shape, with lateral dimensions larger than the height. Mostly, these are synthesized in a circular or elliptical shape, whereas, CNDs are always spherical. CNDs are divided into two subgroups including carbon nanoparticles (CNPs) and carbon quantum dots (CQDs).

**Figure 2:**
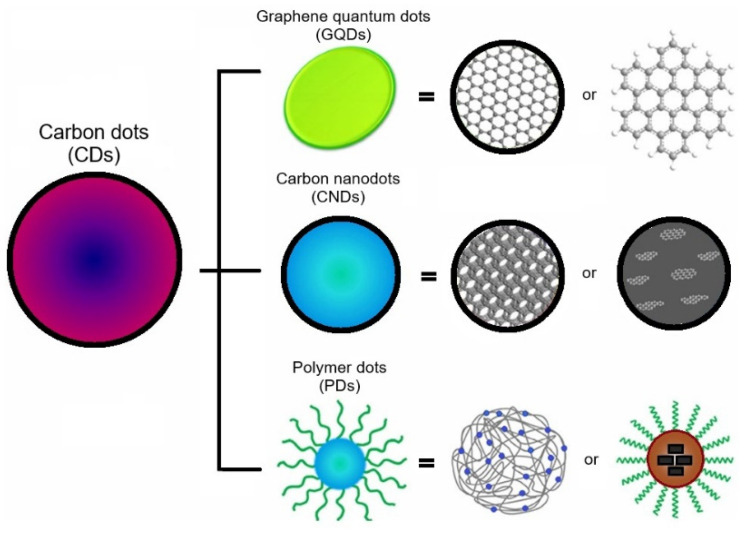
Schematic image of CNDs, GQDs, and PDs structure. Reproduced from ref. [99]. Copyright 2014. With permission from Springer [license no. 5945400714846].

CNPs are amorphous without a clear crystal lattice and do not have any quantum confinement effect (QCE) while CQDs contain an obvious crystal lattice with sp^2^/sp^3^ carbons and have a quantum confinement effect [96,97]. It should be kept in mind that QCE is not dependent on the presence or absence of crystalline structure. When system dimensions are smaller than Bohr’s radius of excitation, conduction and valence bands change from continuous to discrete energy levels restricting the movement of electrons in all directions. This leads to the quantum confinement effect (QCE). When size is higher than 5–7 nm, the particle behaves as a bulk and quantum confinement effects are lost [96]. CNDs have various functional groups on the surface, which play a leading role in the photoluminescence (PL) behavior of these CNDs. These functional groups could be oxygen-based, amino-based groups, polymer chains, etc. PDs are formed by the aggregation or crosslinking of monomers or linear polymers [98,99]. Figure 2 illustrates a typical structure of CNDs, GQDs, and PDs.

One of the most interesting properties that CNDs have is their PL. It has been reported that CNDs are effective in photon-harvesting in the short-wavelength region (260 to 390 nm) because of π−π* transition of C=C bonds and n-π transition of C=O bonds. It is also a known fact that CNDs can emit wavelengths around the whole spectrum of visible light.

However, the PL of CNDs, particularly in GQDs and CQDs, is generally influenced by the QCE and surface states. These states encompass functionalities, defects, heteroatom doping, edge configurations and synthesis methods. The photophysical characteristics of CNDs, stemming from the QCE, are also significantly influenced by particle size. An increase in the size of CNDs leads to a proportional growth in both absorption and emission wavelengths. This size-dependent PL phenomenon is attributed to a reduction in the bandgap, a consequence of π-electron delocalization. Thus, when a particular emission wavelength is desired for CNDs, it necessitates specific shapes and sizes. Achieving this requires a meticulous selection of synthesis methods and precursors that offer control over the synthesis process for CNDs [100,101]. Additionally, the quantum yield (QY) which is related to the emission wavelength is low for CNDs and they are more efficient in absorption of long wavelengths. Whereas, GQDs have higher QYs due to the layered structure and better crystallinity. The QYs of CNDs depend on the synthesis method and carbon source. For instance, CNDs prepared with aliphatic compounds as the carbon source exhibit blue-green luminescence with the QYs of 100% [102], while those prepared with aromatic compounds exhibit yellow emission [103]. CNDs with red emission can be obtained by the addition of acid into the reaction mixture or using a different solvent, such as DMF, while the QYs are very low [104]. However, surface passivation is an efficient technique to improve the QYs and brightness of CNDs [97,105]. The optical properties and PL mechanism of the CNDs are extensively discussed in [95,99,105,106,107,108,109].

### 3.2. Synthesis and Production of Carbon Nanodots

CNDs can be prepared by two main synthetic methods: “top-down” and “bottom-up” methods or in another type of classification, chemical, and physical methods. Each of these methods has some pros and cons which can be chosen regarding the application.

The bottom-up method refers to a synthesis approach where nanomaterials are built by assembling smaller molecular or atomic precursors into larger structures. In this method, small organic molecules or atoms undergo chemical reactions to form complex nanostructures, such as carbon dots (CDs), gradually growing from the “bottom” (small units) to the “top” (complete nanostructure). The bottom-up techniques lie in chemical synthesis and employ pyrolysis and some chemical reactions in order to carbonize small organic molecules. Hydrothermal treatment, microwave, thermal decomposition, template routes, and plasma treatment are examples of bottom-up techniques to produce CNDs in which the precursor shows lower requirements of carbon sources. Bottom-up methods have the advantages of simple surface modification in one-pot and synthesis of variety of structures and functionality of CNDs. However, it is harder to modulate since side products can form that require further purification compared to top-down methods.

The top-down methods, by which CNDs are generally formed through the electrochemical, chemical or physical cutting processes of relatively microscopic carbon structures, such as graphene oxide, carbon nanotubes, graphite powders, etc., and the obtained CNDs resemble the structure of their precursors. The advantages of the top-down methods are the possibility of scaling up the production of CNDs and well-defined CND structures. Top-down methods include laser ablation, electrochemical oxidation, chemical oxidation, and ultrasonic synthesis. In the subsequent sections, a concise introduction to the selected top-down and bottom-up methods is provided.

Laser ablation is a straightforward method for producing CNDs in which carbon precursor is irradiated by a laser. It is environmentally friendly and scalable production method however the energy consumption is high, QY is low, and cannot assure control over the size of the nanoparticle [110,111,112,113]. The nucleation and growth of CNDs consequently its size and morphology depend on the laser pulse width. The CNDs’ size and crystalline grains increased with increasing the laser pulse width [114].

Electrochemical synthesis is another top-down method which can be applied to different bulk carbon materials, such as graphite rod, carbon fiber, and carbon paste. In this method, two carbon-based electrodes are immersed in a water-based electrolyte. By applying redox potential, water electrolysis and H and OH radicals initiate at the edges, and the defects of electrodes act as electrochemical scissors to form CNDs. Similar to laser ablation, this method is also low-cost, scalable, and environmentally friendly, but it has poor size control. However, by tuning the parameters such as applied potential or starting materials CNDs with narrow size distribution can be obtained [115,116]. The major drawback of this method is that it is a tedious purification process, as the obtained CNDs via electrochemical oxidation are highly hydrophilic.

In chemical oxidation, a carbon precursor is oxidized by a strong oxidant to carbon dots. Various oxidants have been used to produce CNDs, such as acids (HNO_3_, H2SO_4_, NaNO_3_, etc.), a mixture of H_2_SO_4_/KMnO_4_/H_2_O_2_ [117], H_2_O_2_ [118] NaOH/H_2_O_2_ [119], and ozone [120]. The oxidation reaction usually requires purification in the case of acidic oxidation in order to remove the acid. Although chemical oxidation is an easy and low-cost method to produce CNDs at a large scale with high QYs, the lack of homogeneity in the size distribution of the resulting CNDs and the risk of burning or explosion are its major disadvantages [121].

Using ultrasonic technology, carbon dots (CNDs) are made by subjecting the reaction mixture to high-intensity ultrasonic waves. This causes cavitation and high-pressure vapor to develop, which facilitates the synthesis of CNDs [122]. This approach presents several benefits, including its affordability, ease of use, and the ability to adjust emission wavelengths to suit different synthesis conditions [123]. However, the efficacy of this method relies on factors such as ultrasonic duration, intensity, and temperature. Variables such as ultrasonic duration, intensity, and temperature can influence various properties of CNDs, including their size, emission wavelength, and surface characteristics. Longer ultrasonic exposure may result in larger CND sizes due to prolonged nucleation and growth, whereas higher intensities can augment cavitation intensity, impacting CND formation [124]. The method’s affordability and simplicity make it a viable option for CND synthesis. Additionally, the ability to adjust emission wavelengths enhances its versatility for different applications [123]. Optimizing ultrasonic parameters can pose challenges, as it may require intricate adjustments and consume time. Furthermore, this technique may lack the precise control over reaction conditions offered by more sophisticated methods [124]. Despite these difficulties, the ultrasonic approach is favored due to its simplicity, cost-effectiveness, and capacity to alter CND characteristics. Although the approach has significant boundaries that may require careful parameter optimization to achieve the desired results, it is nevertheless a popular alternative for creating CNDs. The most common carbon sources for these techniques are carbon nanotubes, activated carbon and graphite.

Hydro/solvothermal methods are widely employed for the synthesis of carbon dots (CNDs), In hydrothermal methods, a water solution of the organic precursor is heated in a sealed reactor, while solvothermal methods use high-boiling organic solvents instead of water, offering better control and improved result [125]. Parameters such as temperature, pressure, precursor concentration, and reaction time significantly influence CND characteristics. Higher temperatures and longer reaction times generally result in smaller CND sizes due to enhanced nucleation and growth kinetics. The advantages of hydrothermal synthesis include its low cost, eco-friendliness, and ability to produce CNDs with tailored properties. However, disadvantages include the requirement for specialized equipment and potential challenges in parameter optimization [126]. Recently, spherical water-soluble CQDs (about 1–3 nm) have been prepared from lemon peel waste using a cost-effective hydrothermal approach. The produced CQDs were observed to have a quantum yield of about 14% and excellent photoluminescence properties with high aqueous stability and oxygen-rich surface functionalities [127]. In another study, fluorescent CQDs (about 260–400 nm) were produced using *Tamarindus indica* leaves via a one-step hydrothermal treatment. These biocompatible CQDs were potentially applied in bio-imaging, disease diagnostics, sensing, and other analytical applications [128]. Citrus lemon juice was also used as a precursor of green synthesis of fluorescent CQDs (2–10 nm), through a one-pot hydrothermal approach. The prepared CQDs exhibited a 10.20% quantum yield. It was reported that the photoluminescence intensity depended on the pH of the solution and maximum intensity was obtained at pH of 6. Synthesized CQDs were also used for cell imaging and compared with the MTT assay to demonstrate its applicability as a fluorescent probe [88]. In another work, amorphous fluorescent CNDs from orange waste peels were produced using the hydrothermal treatment at 180 °C. Consequently, a composite of CNDs with zinc oxide was used as a photocatalyst for the degradation of naphthol blue-black azo dye under UV irradiation, and superior photocatalytic activity was reported [129].

Microwave (MW)-assisted method considered as a green bottom-up technique for CNDs production, providing several benefits including simplicity, speed, and precise control. MW reactors operate on the principles of dipolar polarization and ionic conduction [130,131]. It encompasses a broad spectrum of electromagnetic waves ranging from 1 mm to 1 m, providing accelerated energies ideal for breaking down chemical bonds within precursor molecules. The manipulation of MW parameters significantly impacts the size and attributes of the synthesized CNDs. Adjustments in MW power, synthesis duration, and precursor concentration influence the nucleation and growth kinetics of CNDs, thereby influencing their size distribution [132]. Notably, higher MW power and shorter synthesis durations often yield smaller CND sizes owing to accelerated heating rates and rapid reaction kinetics. Moreover, variations in precursor composition and solvent selection can further adjust CND characteristics, such as fluorescence emission, quantum yield, and surface properties [133]. For instance, it has been observed that the QY of CNDs increased with an increase in MW time, while the QY decreased with the decrease in the MW reaction temperature. MW is a rapid and effective and highly tunable technique for CNDs production. However, the QY is low, and it requires post-modification Moreover, it is high cost and energy consuming. Recently, highly stable and luminescent multicolor CQDs (4.85 ± 0.07 nm) were produced through microwave irradiation of *Cydonia oblonga* powder as a carbon source. Maximum emission intensity at 450 nm was observed for CQDs when it was excited at 350 nm and showed a quantum yield of 8.55% [134].

Pyrolysis/carbonization is considered a bottom-up method for CND production. It involves the decomposition of carbon precursors at high temperatures in the absence of oxygen. Various parameters, such as precursor type, temperature, heating rate, and duration, significantly influence CND size and properties. Higher pyrolysis temperatures typically lead to smaller CND sizes due to increased carbonization efficiency. The advantages of this method include its simplicity, scalability, and the ability to produce CNDs with tailored properties. However, challenges may arise in achieving precise control over CND size and surface functionalities [135].

Other preparation techniques have been also proposed, such as plasma treatment [136], supported synthesis [137,138,139], solution chemistry methods [140,141,142,143], cage-opening of fullerene [144], chemical vapor deposition [145], and template method [146]. The synthesized CNDs typically gain photoluminescence properties via oxidation with nitric acid and surface-passivation via diamine-terminated organic molecules [75]. Interestingly, most of the resulting CNDs exhibit blue or green emissions. The synthesis route and source of carbon are important factors, which affect the size, shape, and properties of the final product. The size of CNDs is very important for understanding quantum phenomena and also for biomedical applications and optoelectronics [107]. Generally, prepared CNDs, regardless of their preparation technique, have a mixture of sizes, which requires complex separation methods to obtain monodisperse CNDs. Some of the post-synthesis separation techniques include dialysis [147], chromatography [148,149] gel electrophoresis [75] and ultra-filtration [150]. Recently, CQDs were prepared by green ozone oxidation of lignite coal, which is abundant and cheap. In this case, also, synthesized CQDs (about 2–4 nm) were observed to be well dispersed in water, contain rich oxygen functional groups and excellent optical properties with a yield of 35%. It was observed that the fluorescence intensity of CQDs has a linear response to the Fe^3+^ concentration varying from 10 to 150 µmol/L. The detection limit was 0.26 µmol/L [119].

### 3.3. Surface Modification and Functionalization of Carbon Nanodots

Surface modification of CNDs is a popular strategy to tailor their properties for specific applications and to improve their performance. Surface functionalization/passivation and doping are two major techniques to chemically modify the surface of CNDs. CNDs’ large numbers of surface functional groups, such as carboxylic groups, hydroxyl groups, amines, etc., make them hydrophilic and facilitate various surface functionalization and passivation. Surface functionalization/passivation of carbon dots refers to the process of treating the surface of CNDs to enhance their stability, biocompatibility, and optical properties. The introduction of various functional groups imposes different defects on the CND surface affecting its excitation energy and leading to large variations in fluorescence emissions. Passivation typically involves the functionalization or coating of the surface of CNDs with various organic, polymeric, inorganic or biological materials. It has several advantages: it prevents the surface defects from which the fluorescence originates; provides reactive sites for surface modification reactions and introduces new properties; enhances the potential application of CNDs for specific sensing, bioimaging, drug delivery, optoelectronics and other specific tasks [151].

The functionalization/passivation strategies can be classified as covalent and non-covalent modification. The covalent modification includes amidation, sulfonylation, esterification, sulfonylation, and copolymerization, while the non-covalent modification includes electrostatic interactions, and π interaction [152]. An effective surface passivation can lead to high fluorescence intensities and high quantum yield of CNDs [112]. Recently, Wang et al. have prepared CQDs with quantum yields of 60% through passivation with PEG_1500N_ [153]. It has been reported that electrochemiluminescence activities depended on the surface passivation of CQDs. Surface passivation reduces the electrochemiluminescence activities and enhances the fluorescence properties [154]. Meanwhile, ultra-small CNDs (0.9 nm) with QY of 47% have been produced through pyrolysis of anhydrous citric acid in organosilane at 240 °C for 1 min. The CNDs functionalized with organosilane can be directly fabricated into a hybrid fluorescent film or monolith via a simple heating process, without using any additional components. It can be further converted into silica-encapsulated NPs by hydrolyzing and co-condensing the CNDs with silica precursors for the biolabeling and imaging applications as well [155]. In another work, a methyl parathion sensor was established from CNDs functionalized with tyrosine methyl ester through hydrothermal reaction using citric acid as a carbon precursor. The functionalized CNDs show strong and stable photo-luminescence with a quantum yield of 3.8%. The obtained biosensors were used for the determination of different kinds of organophosphorus compounds [156]. Hydrothermal treatment of glucosamine produces amino-functionalized fluorescent CNDs. The obtained CNDs displayed stabilized green emission fluorescence at various excitation wavelengths and pH environments which are used to produce biosensors and selective detection of hyaluronidase [157]. In addition to the aforementioned modifying agents, polyethyleneimine [84], boronic acid [158], NH_2_-polyethylene-glycol (PEG_200_) and N-acetyl-_L_-cysteine (NAC) [159] were also used to functionalize CNDs for the application in gene delivery and bioimaging, and blood sugar sensing and Hg (II) sensing, respectively.

**Figure 3:**
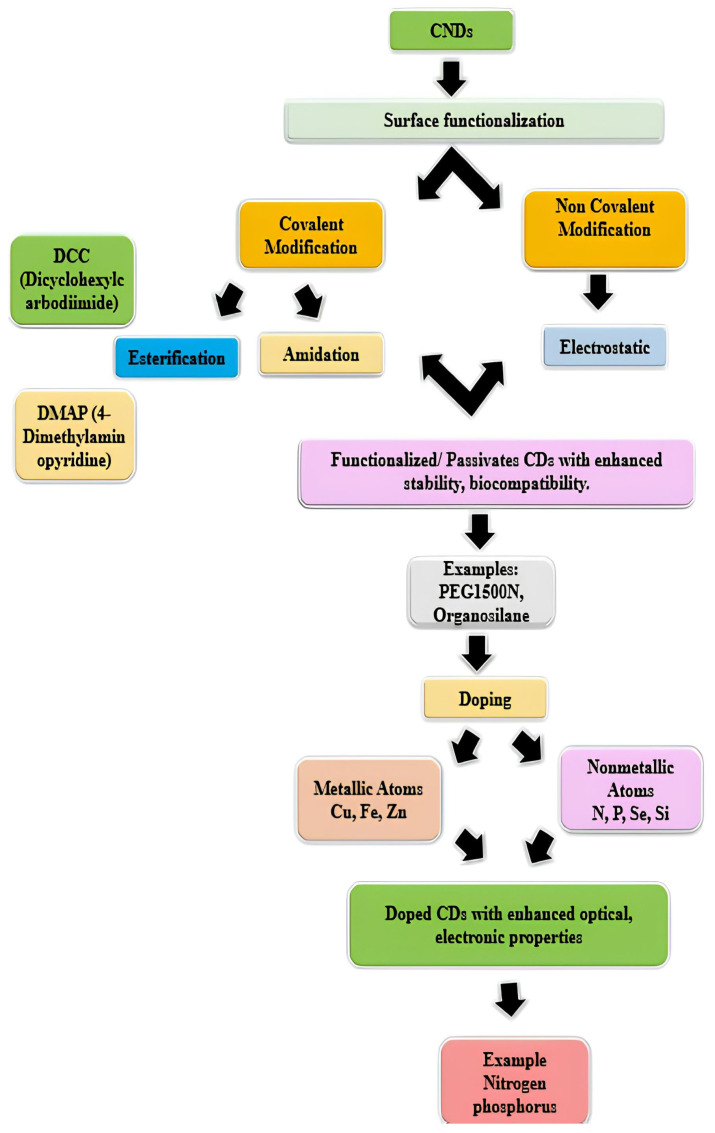
Schematic representation of the chemistry of CNDs.

Doping is another modification technique that involves the introduction of impurities or foreign atoms into the carbon matrix. It offers a powerful means to tailor the properties of CNDs and unlock new functionalities. Atomic doping enhances the florescence performance of CNDs and classified into metallic atoms (such as Cu, Fe, Zn, etc.) or nonmetallic atoms (nitrogen, phosphorus, selenium, silicon, etc.) [160]. Previous studies have demonstrated the effectiveness of doping in enhancing the optical, electronic, and catalytic properties of CNDs. For instance, nitrogen doping has been shown to improve the photoluminescence quantum yield of CNDs, making them more suitable for bioimaging and sensing applications. Similarly, phosphorus doping can enhance the photocatalytic activity of CNDs for pollutant degradation [161]. The advantages of doping include improved stability, enhanced functionality, and tunable properties, which broaden the scope of CND applications in sensing, imaging, drug delivery, and energy conversion. However, doping may introduce defects or alter the surface chemistry of CNDs, potentially affecting their biocompatibility and toxicity. Additionally, factors such as dopant type, concentration, synthesis method, and reaction conditions significantly influence the doping efficiency and resulting properties of CNDs [162]. The schematic representation of the chemistry of CNDs is given below in Figure 3.

## 4. Phytomedicine-Loaded Carbon Nanodots

Carbon nanodots loaded with phytomedicine are a State-of-the-Art example of how nanotechnology and natural medicine can work together to overcome many obstacles in traditional phytomedicine delivery. By utilizing the special qualities of carbon nanodots, this novel synergy improves the bioavailability, targeted administration, and general efficacy of chemicals originating from plants [163].

- Synthesis and Characterization

The process of creating carbon nanodots loaded with phytomedicine is a laborious one that usually uses hydrothermal or other specialized techniques. These nanodots are perfect carriers for phytomedicines because of their unique surface functions and nanoscale size. Carbon nanodots’ surface characteristics can be adjusted to best encapsulate and distribute different kinds of bioactive substances [164].

- Improved Bioavailability

When it comes to overcoming the bioavailability constraints of phytomedicines, carbon nanodots are essential [165]. These nanodots improve the solubility of hydrophobic components and stop them from aggregating and degrading by conjugating or encasing plant-derived chemicals. The enhancement of solubility has a noteworthy impact on the bioavailability of phytomedicines loaded, guaranteeing effective absorption and dispersion throughout the biological system [166].

- Targeted Drug Delivery

One of the main benefits of personalized medicine is the ability to precisely target particular tissues or cells through the functionalization of carbon nanodots [167]. By interacting with particular biomolecules or receptors, surface changes let the loaded phytomedicines reach their targeted sites of action. By reducing adverse effects and off-target consequences, this focused administration maximizes therapeutic efficacy.

- Controlled-Release Kinetics

Carbon nanodots’ controlled-release properties elevate the delivery of phytomedicine to a higher level. These nanodots allow for a controlled and prolonged release of the loaded phytomedicines by varying the surface characteristics or adding responsive components. By preserving ideal medication concentrations for prolonged periods of time, this controlled-release profile improves therapeutic effects and may lessen the frequency of administration [168].

- Biocompatibility and Safety

Because of their low toxicity and great biocompatibility, phytomedicine-loaded carbon nanodots are safe to use in biological systems. The effects of these nanodots on cellular architecture, metabolic pathways, and general physiological functions are the subject of much research [169,170]. Carbon nanodots’ biodegradable nature adds to their safety profile and allays worries about long-term exposure.

- Intracellular Delivery and Cellular Uptake

Enhancing cellular absorption is a critical component for intracellular targets, and carbon nanodots’ nanoscale size makes this possible. It is possible to effectively transport phytomedicines over cellular barriers, guaranteeing their delivery to targeted cell compartments. The greater potency and overall bioavailability of the loaded phytomedicines are a result of this increased cellular absorption [171].

- Encapsulation of Diverse Phytochemicals

Carbon nanodots loaded with phytomedicine are adaptable vehicles that can hold a wide variety of phytochemicals, such as polyphenols, terpenoids, alkaloids, and flavonoids. This adaptability broadens the application of this delivery technology across different herbal medicines by enabling the encapsulation of complex mixes present in medicinal plants [172].

- Application in Multicomponent Phytomedicine Formulations

Phytomedicine-loaded carbon nanodots present an opportunity to formulate complicated, multicomponent delivery systems in the context of traditional herbal treatments that contain multiple active components. By delivering synergistic plant-derived components simultaneously, this method can enhance therapeutic effects and replicate the holistic aspect of conventional herbal preparations.

- Future Perspectives and Challenges

Natural therapies could undergo a revolution with the introduction of carbon nanodots loaded with phytomedicine into the medication delivery system. Research is still ongoing in several areas, including scalability, standardized synthesis techniques, and long-term safety assessments. Subsequent efforts will concentrate on optimizing these delivery systems based on nanodots for broad clinical use, with a focus on regulatory compliance and reproducibility [173]. To sum up, the creation of carbon nanodots loaded with phytomedicines is a cutting-edge in the field of sophisticated medication administration. With its capacity to target delivery, ensure regulated release, and overcome bioavailability limitations, this novel technique has enormous promise to further the medicinal applications of chemicals produced from plants. The current research in this area marks the beginning of a new era in which the combination of natural medicines and nanotechnology creates opportunities for improved treatment results.

### 4.1. Loading Strategies for Phytomedicine

Recent studies have thrown light on novel strategies and developments in this emerging field, offering insightful information about phytomedicine loading tactics onto carbon nanodots. Modern technologies and a greater comprehension of the interactions between nanomaterials have led to the adaptation and improvement of these tactics, which are essential for maximizing the effectiveness of medicine administration.

- Physical adsorption: recent developments

Current research has looked into creative approaches to improve phytomedicine’s physical adsorption onto carbon nanodots [11]. Through specialized chemical interactions, surface modification of nanodots with particular functional groups, including amino or hydroxyl groups, has been studied to increase adsorption affinity [174]. Furthermore, sophisticated characterization methods have been used to accurately comprehend and control the non-covalent forces regulating adsorption, such as spectroscopic studies and computational modeling. The goal of these improvements is to get over issues with adsorption strength variability and environmental sensitivity.

- Covalent bonding: progress in precision

The goal of covalent bonding strategy advancements has been to increase loading-process precision. Researchers have looked into site-specific functionalization of carbon nanodots to facilitate selective covalent interaction with certain areas of phytomedicine molecules [175]. This focused strategy reduces inadvertent changes and maintains the integrity of the loaded chemicals. Furthermore, current studies have focused on the creation of green chemistry strategies that maximize covalent bonding without sacrificing the phytomedicine’s therapeutic qualities [176]. These strategies make use of environmentally friendly solvents and reaction conditions.

- Encapsulation during nanodot synthesis: tailoring synthesis conditions

Recent developments in the field of encapsulation during nanodot synthesis have focused on optimizing synthesis conditions for enhanced control. Mathew et al. prepared chitosan/carbon dots to be used as a drug carrier for neurodegenerative diseases. Encapsulation of a phytomedicine in a carbon nanodot can be performed with the help of entrapment of drug in a polymer matrix and further encapsulating the drug–polymer matrix into a carbon nanodot [177]. Novel techniques like template-assisted procedures and microfluidic-assisted synthesis have been investigated to obtain a more accurate phytomedicine compound encapsulation. These methods address issues related to consistent distribution and possible changes in the therapeutic qualities of loaded phytomedicine during synthesis, providing scalability and reproducibility. Hollow CNDs can encapsulate the phytodrugs through hydrophobic and electrostatic interactions, pi-pi stacking, or hydrogen bonding, which needs more research for validation [35].

### 4.2. Stability and Bioavailability Enhancement

Recent work on phytomedicine-loaded carbon nanodots has yielded important advances to improve the stability of encapsulated bioactive chemicals, as well as novel approaches to increase bioavailability. These developments, which are based on cutting-edge techniques and technologies, provide an early look at more reliable and effective medication delivery methods in the future.

- Enhanced stability through nanoengineering

Utilizing cutting-edge nanoengineering methods to increase the stability of carbon nanodots loaded with phytomedicine has been the focus of recent research. Researchers have investigated how to produce protective shells around the nanodots by nanoencapsulation techniques, such as lipid-based formulations and polymeric coatings, to shelter the encapsulated phytomedicine from environmental stressors [178]. Furthermore, surface alterations like the application of silica or graphene oxide coatings have shown improved stability, delayed premature deterioration and maintained the bioactivity of the loaded chemicals.

- Smart nanomaterials for controlled release

Recent studies have investigated the incorporation of smart nanomaterials into carbon nanodots as a means of achieving controlled and sustained release mechanisms. The creation of intelligent drug delivery systems has sparked research into stimuli-responsive nanocomposites, such as pH-sensitive polymers and temperature-responsive hydrogels. These technologies provide customized and on-demand delivery of phytomedicine components by precisely controlling release kinetics in response to particular physiological situations.

- Advances in nanotoxicology and biocompatibility

Recent study has focused on ensuring the safety and biocompatibility of carbon nanodots loaded with phytomedicines. Utilizing cutting-edge imaging methods, including live-cell imaging and super-resolution microscopy, advanced nanotoxicology investigations have thoroughly evaluated the interactions between nanodots and biological systems at the cellular and subcellular levels [179]. This thorough knowledge has facilitated the creation of safer nanomaterials, which may reduce the possibility of cytotoxic effects and open the door to their use in biomedicine.

- Precision bioavailability enhancement

Improvements in lipid nanoparticle and micelle technologies have served as an inspiration for the development of nanoscale drug delivery vehicles, which have shown to better solubilize hydrophobic phytomedicine components and increase their bioavailability [180]. To further maximize bioavailability, studies have also looked at the use of ligand-targeted nanocarriers, which take use of ligand–receptor interactions to enable tailored distribution and uptake.

- Emerging trends and future directions

Trends in stability and improving bioavailability are moving in the direction of a multidisciplinary strategy. The amalgamation of computational modeling, artificial intelligence, and systems biology is increasingly common in order to forecast and enhance the behavior of nanomaterials in intricate biological contexts. Biosensors and imaging modalities are among the real-time monitoring approaches that are being used to offer dynamic insights into the in vivo fate of carbon nanodots loaded with phytomedicine. Another well-known trend in nanotechnology is the hunt for “green” materials. To address sustainability issues, researchers are investigating biodegradable nanomaterials and ecologically friendly synthesis techniques [181]. The goal of surface modification innovations like bioinspired coatings and biomimetic methods is to produce nanocarriers that are less immunogenic and more biocompatible. As the field advances, it is anticipated that these recent research insights and technological innovations will propel phytomedicine-loaded carbon nanodots towards broader clinical applications, offering more effective, safe, and targeted therapeutic interventions.

### 4.3. Controlled-Release Mechanisms

With a focus on accuracy, flexibility, and responsiveness, recent research has made significant advancements in the field of controlled-release mechanisms for carbon nanodots loaded with phytomedicine. The field of controlled drug delivery is being shaped by cutting-edge technologies and creative approaches, which provide insights into how nanotechnology might be used to maximize therapeutic results.

- Advanced surface engineering for tunable release

In order to give carbon nanodots variable releasing capabilities, recent investigations have investigated sophisticated surface engineering techniques [182]. Dynamic changes in release kinetics are possible by functionalizing the nanodot surface with stimuli-responsive polymers or biomimetic coatings. The focus has been on pH-responsive systems in particular, as they allow for controlled release in response to variations in pH that occur in various physiological compartments. The accuracy attained with these surface alterations guarantees a customized release profile in line with therapeutic requirements.

- Microfluidic-assisted synthesis for controlled encapsulation

One notable development in the field of encapsulation techniques is microfluidic-assisted synthesis. By allowing for exact encapsulation of phytomedicine components within carbon nanodots, this approach offers tight control over the production process. The continuous and uniform mixing of reactants made possible by microfluidic devices produces nanodots with consistent characteristics [183]. This degree of control makes drug-loaded nanodots more reproducible and helps to ensure a more regulated and predictable release of phytomedicine.

- Temperature-responsive systems for on-demand release

Temperature-responsive components incorporated into carbon nanodots have been the subject of recent studies, allowing for on-demand medication delivery [184]. These devices use the natural temperature fluctuations that occur in physiological settings to initiate regulated release. It is now possible to adjust the rate of drug release in nanodot formulations in response to particular thermal cues by using thermoresponsive polymers or materials that are susceptible to external heat sources. This strategy has potential for use in situations where exact control over release dynamics is essential.

- Advancements in biodegradable nanocarriers

A crucial factor in the development of controlled-release systems is biodegradability. The creation of biodegradable nanocarriers that can deliver phytomedicine in a regulated manner as they decompose over time has been the focus of recent developments. Researchers have looked into using biocompatible materials, including certain polymers and lipid-based nanoparticles, to make nanocarriers that break down gradually. This allows the release kinetics to be in line with therapeutic requirements and reduces the possibility of long-term accumulation issues [185].

- Emerging trends and future prospects

A convergence of disciplines characterizes emerging developments in controlled-release mechanisms for carbon nanodots loaded with phytomedicine. Predicting and optimizing release patterns based on a variety of criteria, such as pharmacological qualities, patient-specific data, and nanomaterial features, is becoming more popular by integrating nanotechnology with AI and machine learning [186].

In addition, the latest developments in real-time monitoring methods, such biosensors and in vivo imaging, provide dynamic insights into the spatiotemporal behavior of nanodots inside the body [187]. This real-time feedback loop makes adaptive release strategy modifications possible, improving the efficacy and adaptability of controlled drug delivery systems.

**Figure 4:**
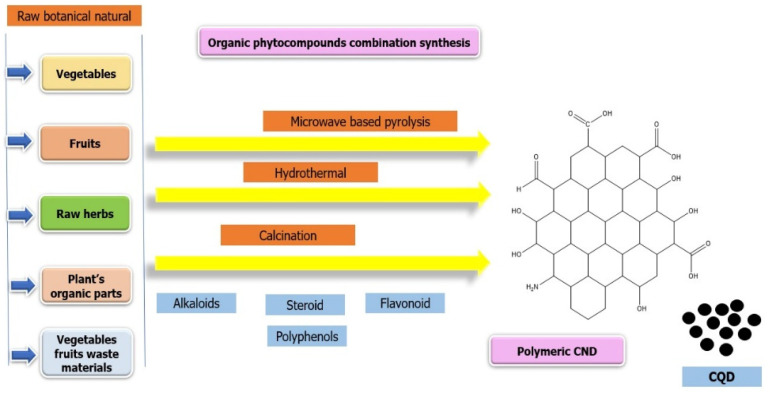
Overview of synthesis of CND from plants, with raw materials as precursors.

Emerging trends in the field suggest that regulated release mechanisms for carbon nanodots loaded with phytomedicines may change as a result of these recent discoveries and technological advancements. This will present prospects for personalized and precision medicine with improved therapeutic precision. An overview of the synthesis of CNDs from plants and raw materials as precursors is shown in Figure 4.

## 5. Polymeric Carbon Nanodots

### 5.1. Introduction to Polymer Nanocomposites

Over the past two decades, considerable scientific and technological interest has centered on polymer nanocomposites (PNCs). Initially, the focus was primarily on understanding synthetic and physical chemistry, colloidal particle physics, and various properties of these materials. Recently, there has been a shift towards comprehending and harnessing the unique physics of polymers within PNCs. This shift is driven by the realization that achieving truly engineered and functional nanocomposites requires a deeper understanding of their structure–property–processing relationship. Modern developments in the realm of nanomaterials include polymeric carbon nanodots (CNDs) and polymer nanocomposites, each of which offers a distinct set of qualities and uses. Since they are so adaptable, polymeric CNDs which are essentially nanoscale carbon-based particles have attracted a lot of attention [188]. They also have unique characteristics like high photoluminescence. However, by adding nanoscale fillers, such as nanoparticles or nanotubes, to polymer matrices, material characteristics can be improved, as in the case of polymer nanocomposites. Superior mechanical, thermal, and electrical properties are added to the resultant composite materials by the interaction of polymers and nanofillers [189]. Polymeric CNDs are distinguished by their distinct composition and structure. A majority of the carbon atoms in the core are grouped either crystalline or amorphously, resembling graphene. Functional groups including hydroxyl, carboxyl, and amino groups are present on the surface of these nanodots, which enhances their solubility, stability, and reactivity. The size and characteristics of the nanodots are affected by various techniques used in the synthesis of polymeric CNDs, including hydrothermal synthesis, microwave-assisted synthesis, and template-assisted synthesis [190]. Conversely, to produce materials with improved performance, polymers are blended with nanoscale fillers to make polymer nanocomposites. When nanofillers are added, the material behaves differently from a pure polymer, providing increases in stiffness, strength, and other important characteristics. Polymeric CNDs are distinguished by their distinct composition and structure. A majority of the carbon atoms in the core are grouped either crystalline or amorphously, resembling graphene [191]. On the other hand, polymer nanocomposites comprise a wider range of materials in which nanofillers enhance overall performance and polymers function as matrices. The addition of nanofillers improves the composite’s mechanical, thermal, and electrical qualities, making it appropriate for use in the automotive, electronics, and aerospace industries. Melt blending, solution mixing, and in situ polymerization are the synthesis techniques used to create polymer nanocomposites; each has an impact on the properties of the finished product [192].

### 5.2. Structural Composition

Polymer nanocomposites’ structural elements are distinguished from traditional composites by the accurate dispersion of nanofillers inside the polymer matrix. In this nanoscale configuration, the polymer matrix is finely interwoven with nanofillers, which can be nanoparticles, nanotubes, or other nanomaterials. Since it has a significant impact on the composite material’s overall performance, this careful dispersion is essential. It is a laborious process with major consequences to distribute nanofillers uniformly throughout the polymer matrix. Given that they are nanoscale, the nanofillers are smaller than those that are usually employed in traditional composite materials. Its reduced size makes it possible for the polymer to be more thoroughly and uniformly integrated, which enhances its desirable qualities. A uniform dispersion of the nanofillers highlights the enhanced attributes, which include greater stiffness, strength, and thermal stability. This structural configuration at the nanoscale contrasts sharply with traditional composites, where the fillers are frequently bigger and less uniformly distributed. Uniform dispersion is more difficult to achieve in typical composites, and the unequal distribution of the filler elements can lead to variances in the properties of the final material. By optimizing the advantages of the nanofillers and enhancing the material’s overall performance, polymer nanocomposites’ nanoscale accuracy guarantees that the improved properties are consistently manifested throughout [193].

### 5.3. Various Synthetic Methods of Polymeric Carbon Nanodots

**Figure 5:**
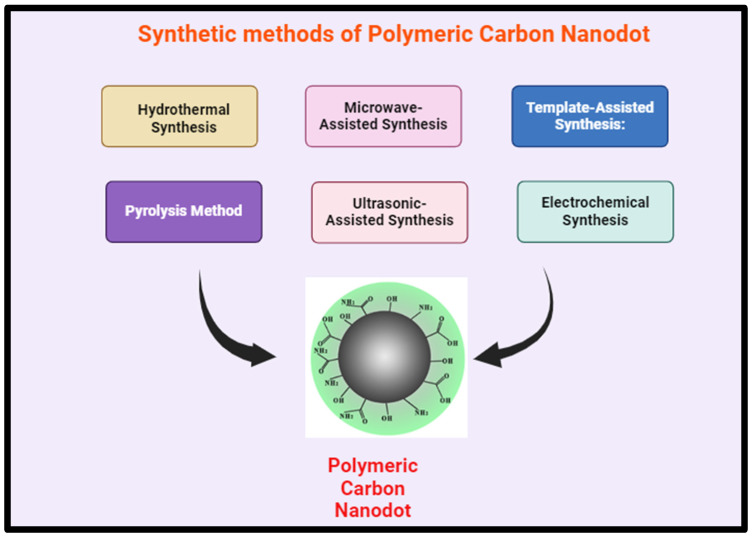
Various methods of synthesis of polymeric carbon nanodots.

The process for generating polymeric carbon nanodots (CNDs) involves a range of techniques designed to produce carbon particles at the nanoscale with particular characteristics. Many well-established methods are used to synthesize these polymeric CNDs, which are preferred for their improved stability, biocompatibility, and flexible functionalization potential. By using microwave irradiation to speed the reaction kinetics, however, microwave-assisted synthesis produces polymeric CNDs with regulated characteristics quickly and effectively. Using templates like polymers or proteins, template-assisted synthesis allows for fine control over the morphology of the CNDs during production by dictating their size and shape [194]. Pyrolysis is a versatile process that uses a variety of carbon-rich sources and offers sustainability by carbonizing organic precursors which are often sourced from biomass under regulated circumstances. Using ultrasonic vibrations, bigger carbon structures can be gently and energy-efficiently broken down into nanoscale CNDs through the process of ultrasonic-assisted synthesis. With electrochemical synthesis, one can precisely regulate the synthesis conditions by inducing the creation of polymeric CNDs with the use of electrical energy [195]. Lastly, under benign and eco-friendly circumstances, photochemical synthesis uses light as an energy source to start reactions that result in the synthesis of polymeric CNDs. The resultant polymeric CNDs are adaptable for a variety of applications, including biological imaging, sensors, and optoelectronics, and can be further characterized and functionalized. The desired characteristics and planned uses of the polymeric CNDs inform the synthesis technique selection, which reflects the dynamic and varied nature of the nanomaterial sector. Various methods of synthesis of polymeric carbon nanodots are shown in Figure 5.

#### 5.3.1. Role of Polymer Nanocomposites in Drug Delivery

By addressing issues with traditional drug delivery systems, these nanocomposites, which are made of polymers and nanoscale materials, have opened the door for more accurate and regulated drug release. The potential of polymer nanocomposites to encapsulate and shield medications, preventing their untimely breakdown or excretion from the body, is one of its main benefits. This is especially important for medications that are difficult to degrade in an adverse biological environment or have low bioavailability. The medication is protected from the environment by nanocomposites, which also guarantee its stability until it reaches the intended location [196]. It is possible to modify the drug delivery system’s characteristics by adding elements at the nanoscale, like nanoparticles, to polymer matrices. Some features, including targeting, can be built into these nanoparticles to allow the nanocomposites to selectively aggregate at the sick tissue while sparing healthy cells. By minimizing systemic side effects, this focused drug administration improves the overall therapeutic result [197]. In addition, these composites’ nanoscale size makes it easier for them to pass across biological barriers like blood–brain barriers and cell membranes. This makes it possible to administer medications to regions that were previously unreachable or difficult to treat, so broadening the range of illnesses that can be treated, including neurological conditions. In order to achieve controlled and prolonged medication release, polymer nanocomposites are also essential [198]. The release kinetics of the medication enclosed can be precisely tailored by researchers by carefully crafting the polymer matrix and modifying the composition of the nanoparticles. This helps maintain therapeutic concentrations over an extended length of time, lowering the frequency of dose and increasing patient compliance. It is especially helpful for chronic illnesses that call for continuous and controlled drug administration. The capacity of polymer nanocomposites to encapsulate a broad variety of medicinal substances, such as proteins, nucleic acids, and tiny molecules, is another example of their versatility [19]. The creation of multifunctional drug delivery devices that can administer combination medicines is made possible by this flexibility. For example, a nanocomposite may carry a gene therapy payload and a chemotherapeutic medication at the same time, resulting in a synergistic effect that improves treatment outcomes. Another crucial component of polymer nanocomposites in drug administration is their biodegradability [199]. The fact that a lot of these polymers break down gradually into inert byproducts helps to prevent the delivery system from building up within the body. This feature is especially helpful since it reduces the possibility of long-term side effects and permits the carrier to be cleared after its medication-delivery purpose is completed. Drug delivery has advanced even further with the creation of stimuli-responsive polymer nanocomposites. These intelligent materials are able to react to particular bodily stimuli, such as variations in pH, temperature, or the presence of enzymes. This responsiveness improves accuracy and lessens off-target effects by enabling triggered medication release at the target spot [200]. For example, a medicine encapsulated in a nanocomposite that is made to withstand the acidic environment of tumors can release the drug only in the malignant tissue. Additionally, polymer nanocomposites have shown great promise in addressing drug resistance, a critical obstacle in the treatment of several illnesses, most notably cancer. These composites’ multifunctionality enables the co-delivery of medications with various modes of action, addressing several drug resistance routes and raising the possibility of successful therapy. To sum up, polymer nanocomposites have a revolutionary function in drug delivery and provide a host of benefits over conventional drug delivery methods. These nanocomposites offer a platform for accurate, focused, and managed medication delivery. Future customized and extremely successful therapeutic interventions appear to be in the cards as this field of study progresses and more complex polymer nanocomposites are created [201].

#### 5.3.2. Properties of Polymeric Carbon Nanodots

Polymeric carbon nanodots have outstanding photoluminescence, which is one of their prominent features. In the presence of visible or ultraviolet light, these nanodots can release visible light. Because CNDs emit light and can be adjusted in intensity, this feature is especially useful for bioimaging and fluorescence imaging applications, which require sensitive and accurate identification of biological processes and structures. Additionally, the capacity to regulate the emission wavelength. When these nanodots are subjected to ultraviolet or visible light, they can release visible light. The strong and adjustable emission of CNDs makes it possible to precisely and sensitively identify biological structures and processes, making this trait very beneficial for applications in fluorescence imaging and bioimaging [202]. Their further usefulness in multicolor imaging and diagnostics is increased by the controllable emission wavelength. Biocompatibility is another well-known characteristic of polymeric CNDs, which is essential for biomedical applications. Their composition guarantees low cytotoxicity and immunogenicity; they are frequently made from biocompatible polymers or carbon precursors [203]. Because of this, they can be used in a variety of biological settings, such as medication delivery, imaging in vivo and in vitro, and other biomedical applications. Polymeric CNDs have the potential to be safe and useful medical instruments because of their biocompatibility. The adaptable surface chemistry of polymeric carbon nanodots is another significant characteristic. It is simple to alter or functionalize the surface of CNDs with different groups, such as carboxyl, amino, or hydroxyl groups. The CNDs can be given extra functions by attaching particular ligands, biomolecules, or medications due to their adjustable surface chemistry. Functionalized polymeric CNDs exhibit great adaptability to individual requirements, as they can be customized for specialized applications like sensing, targeted medication delivery, or other uses [204]. Excellent water solubility is another feature of polymeric CNDs that makes them useful for biological and environmental applications. Because of their hydrophilic character, these nanodots are easily incorporated into biological systems by dispersing in aqueous solutions. This solubility is especially useful in drug administration applications, where stable physiological conditions and effective dispersion are essential for effective therapeutic results. Moreover, polymeric CNDs show stability under a range of environmental circumstances. Because of their resilience, they can tolerate variations in pH, temperature, and light exposure, thus guaranteeing their performance and functioning over time. The practical applications of CNDs in a variety of sectors, from electronics to biology, depend on this stability [205]. One of the notable characteristics that helps explain polymeric carbon nanodots’ consistent and predictable performance is their homogeneity in size and shape. The manufacturing of nanodots with precise sizes and shapes is made possible by the controlled synthesis techniques used in their creation [206]. For uses like catalysis, sensing, or electrical devices where exact control over the characteristics of nanomaterials is critical this uniformity is vital [207]. To sum up, polymeric carbon nanodots have a number of amazing qualities that make them very appealing for a variety of uses. Their promise in several sectors like materials science, biomedical research, and sensing technology is enhanced by their photoluminescence, stability, water solubility, biocompatibility, and size uniformity. The special qualities of polymeric CNDs are anticipated to open up even more creative uses as research in this field develops, solidifying their status as useful nanomaterials across a wide range of scientific and technical fields [208].

### 5.4. Applications of Polymeric Carbon Nanodots in Drug Delivery

As a result of their distinct qualities and range of functions, polymeric carbon nanodots (CNDs) have become a viable nanomaterial for drug delivery applications. The use of polymeric CNDs in drug delivery has been discussed, along with their applications and underlying mechanics.

Applications for Drug Delivery Using Polymeric Carbon Nanodots:

- Delivery of Drugs with Specificity:

To distribute drugs in a targeted manner, polymeric CNDs can be functionalized with certain ligands, including peptides or antibodies. Target cells or tissues’ surface receptors are the only receptors that the functionalized nanodots can bind to with selectivity. The tailored strategy improves overall treatment efficacy, minimizes off-target effects, and concentrates the therapeutic payload at the intended site to increase drug delivery efficiency [209].

- Imaging and Diagnosis:

Polymeric CNDs are perfect for imaging and diagnostic applications due to their exceptional photoluminescent characteristics. CNDs can allow for real-time medication delivery process monitoring when they are utilized as imaging agents. Furthermore, the vivid and adjustable fluorescence of CNDs can help see biological structures, which can help with illness monitoring and diagnostics [23,208].

- Theragnostic:

The potential for polymeric CNDs to integrate therapeutic and diagnostic functions onto a single platform is known as their theragnostic potential. Researchers can create systems that not only carry medications to the target region but also provide real-time feedback on the effectiveness of the treatment by combining therapeutic chemicals and imaging probes into the CNDs. Precision therapies and personalized treatment will be greatly impacted by this integrated approach [199].

- pH-Responsive Drug Release:

It is possible to incorporate pH-responsive components into polymeric CNDs due to their changeable surface chemistry. CNDs can undergo controlled disintegration or structural changes that release medications that are contained in acidic environments, like those seen in tumor tissues. By maximizing the delivery of therapeutic payloads to sick tissues and reducing exposure to healthy cells, this pH-responsive behavior improves drug release precision [210].

- Combination Therapy:

Combination therapy is made possible by the platform that polymeric CNDs provide, which enables the simultaneous loading of several therapeutic drugs with various modes of action. When treating complex diseases like cancer, where a combination of medicines may be more effective in reducing drug resistance and improving treatment results, this is very helpful.

- Intracellular Delivery:

Drug distribution into cells is made easier by the nanoscale size of CNDs, which allows them to pass through cell membranes. This characteristic makes medications that target intracellular processes more effective in treating patients by enabling the delivery of medications to particular cellular compartments or organelles.

In order to transfer stem cells to diabetic wounds, a novel pH and thermosensitive hydrogel made of chitosan (CTS) and sADM modified with carbon nanodots (CND) from onion peels is presented in the work by Bankoti et al. where enhanced diabetic wound healing can be achieved with the help of this hybrid hydrogel’s several advantages, which include enhanced hydrophilicity, sustained release of ND, antibacterial and antioxidant qualities, promotion of stem cell delivery, and excellent biocompatibility and biodegradability. In addition to stimulating angiogenesis and scavenging reactive oxygen species (ROS), the hydrogel containing ND also makes it easier for stem cells to be encapsulated and delivered to the wound site, hastening wound closure and encouraging tissue regeneration without the formation of scars. In addition, the hydrogel has a moderate level of cytocompatibility and antimicrobial activity, which guarantees low cytotoxicity and supports cell viability. The study highlights the potential of capped or biopolymer-doped carbon nanodots in biomedical applications, especially advanced wound healing, where their multifunctional properties are crucial for tackling the intricate issues related to diabetic wounds, ultimately resulting in improved clinical outcomes [211].

### 5.5. Polymeric Carbon Nanodots’ Mechanisms of Drug Delivery

Targeting passively: The Enhanced Permeability and Retention (EPR) effect, which is seen in tumors where leaky vasculature permits the preferential accumulation of nanoparticles, can be exploited by polymeric CNDs. CNDs can accumulate at the tumor site through passive targeting, which increases the amount of medication delivered to cancer cells.

Active targeting: Targeting ligand-functionalized polymeric CNDs allows active targeting. Ligands, which are recognized and bind to specific receptors on the surface of target cells, can be peptides or antibodies. This process facilitates the internalization of CNDs and the subsequent release of therapeutic payloads. By improving drug delivery’s specificity through active targeting, systemic adverse effects are decreased.

Drug release that is pH-responsive: Polymeric CNDs have the ability to react to pH variations. It is possible for CNDs to experience structural alterations that result in the release of encapsulated medications in the acidic environment of tumors or certain cellular compartments. The accuracy and effectiveness of treatment are enhanced by this pH-responsive behavior, which guarantees that drug release is initiated at the targeted spot [212].

Cellular uptake and endocytosis: Polymeric CNDs’ interactions with cells are influenced by their surface characteristics and nanoscale size. Different endocytic mechanisms, including caveolae- or clathrin-mediated endocytosis, can be used to internalize CNDs. CNDs’ efficiency in intracellular drug delivery is attributed to their capacity to man oeuvre these cellular processes.

Sustained release: The medicine enclosed in CNDs can be released gradually due to the polymer matrix’s architecture. Researchers can attain a regulated and extended-release profile by modifying the drug’s interactions with the polymer or the polymer’s rate of breakdown. When treating long-term illnesses that call for constant medication administration, this sustained release method is especially helpful [213].

Intracellular trafficking: Polymeric CNDs go through intracellular trafficking after being absorbed by cells. In order to maximize the effects of drug delivery, it is essential to comprehend the routes and destiny of CNDs within cells. Drug delivery can provide a greater therapeutic effect by optimizing drug release at certain subcellular sites through manipulation of CND intracellular trafficking.

To sum up, polymeric carbon nanodots have proven to have great promise for drug delivery applications. They provide a flexible platform for the precise and regulated release of medicinal molecules. Polymeric CNDs have distinct qualities like photoluminescence, biocompatibility, and programmable surface chemistry along with well-defined drug delivery systems that make them attractive options for furthering the science of nanomedicine. More advancements in the creation and use of polymeric CNDs are anticipated as this field of study develops, perhaps leading to the creation of more specialized and efficient drug delivery systems [214].

### 5.6. Polymeric Carbon Nanodots’ Advanced Properties and Functionalities

Polymeric carbon nanodots (CNDs) display a variety of complex properties that go beyond simple photoluminescence, making them very desired in domains that need multifunctional and flexible materials. Polymeric CNDs’ outstanding characteristics result from their nanoscale size, unique carbon-based architecture, and surface functionalization, which increase their applicability across disciplines [201].

#### 5.6.1. Improved Photostability and Tunable Fluorescence

Polymeric CNDs differ from typical fluorescent dyes in terms of photostability, namely photobleaching resistance. Unlike organic fluorophores, which decay after prolonged exposure to light, polymeric CNDs retain their fluorescence via several excitation cycles. This makes them perfect for long-term imaging in biological and environmental settings [215]. Furthermore, scientists may control the fluorescence emitted by polymeric CNDs by altering synthesis parameters, surface functional groups, and dopants, allowing them to generate a wide range of colors. This tunability offers significant advantages for developing multicolor probes for sophisticated bioimaging and optical sensing applications [201].

#### 5.6.2. Electronic Properties and Conductivity

Polymeric CNDs have unique electrical characteristics due to their conjugated π-electron systems, including semi-conductivity and high electron mobility. These properties, particularly in CNDs synthesized with graphitic structures, make them ideal for optoelectronic applications such as solar cells, LEDs, and photodetectors. Researchers can further alter the electronic characteristics of carbon frameworks by including heteroatoms like nitrogen, sulfur, or boron, leading in CNDs customized for certain electrical or catalytic functionalities [216]. Conductivity of CNDs helps in better interaction of drug-loaded CNDs with biological membranes, leading to improvement in release profile, as well as bioavailability [30].

#### 5.6.3. Catalytic Activity and Environmental Applications

Research demonstrates that polymeric CNDs can work as catalysts or catalytic supports due to their large surface area, functionalizable surfaces, and capacity to effectively transport electrons. For example, nitrogen-doped CNDs have a high catalytic potential in oxygen reduction processes, which are important in fuel cell technology. In a similar way polymeric CNDs may help in the photocatalytic destruction of contaminants, providing a sustainable method to wastewater treatment [217,218]. Furthermore, the catalytic properties of CNDs enable it to scavenge reactive oxygen species with ease. This compliments the organ-protective abilities of phytomedicine-derived CNDs, which are advantageous for developing therapies especially for liver conditions and neurodegenerative disorders [2,219].

### 5.7. Enhanced Synthetic Approaches for Polymeric Carbon Nanodots

Beyond standard hydrothermal and microwave-assisted synthesis, novel and hybrid synthesis techniques are being developed to improve the characteristics and functions of polymeric CNDs.

#### 5.7.1. Ultrathin Layered Synthesis

Ultrathin layered synthesis is a new approach for producing CNDs with precisely controlled thickness at the nanoscale. Under regulated circumstances, scientists may layer carbon precursors on substrates like silica or alumina to create ultra-thin CND layers with unique optical characteristics due to quantum confinement. This approach is especially beneficial in optoelectronics and biosensing, where precise control of CND thickness is required to tune electrical and photonic behavior [220].

#### 5.7.2. Dopant-Driven Synthesis Techniques

Dopant-driven synthesis approaches have evolved that insert heteroatoms or functional groups into CND structures during the synthesis process rather than afterward. CNDs can be designed to have enhanced fluorescence, conductivity, or catalytic activity by adding nitrogen, boron, or sulfur during the initial synthesis stages. Dopant-driven techniques are especially useful in electrochemistry and catalysis, where heteroatom-rich CNDs outperform pure carbon equivalents [221].

#### 5.7.3. Green Synthesis Using Biomass Precursors

Agricultural waste, plant extracts, and even food scraps are plentiful and sustainable sources of carbon. These green synthesis processes are not only cost-effective, but also waste-reducing, making them appealing for large-scale manufacturing. Furthermore, biomass-derived CNDs frequently demonstrate built-in biocompatibility, which increases their suitability for biomedical applications [222].

### 5.8. Functionalizing Polymeric Carbon Nanodots for Specific Applications

#### 5.8.1. Surface Functionalization for Targeted Drug Delivery

Surface-functionalized CNDs can be modified with peptides, antibodies, or other biomolecules to improve medication targeting sick cells or tissues. By conjugating targeting ligands, CNDs may recognize and bind to specific cell receptors, enabling selective accumulation at certain areas, such as tumor tissues. This ligand-mediated targeting lowers off-target effects, systemic toxicity, and increases the therapeutic effectiveness of the drug payload [34].

#### 5.8.2. Polymer Coatings for Improved Biocompatibility and Stability

Coating CNDs with biocompatible polymers such as polyethylene glycol (PEG) or polylactic acid (PLA) increases their stability and biocompatibility. Because of the “stealth” effect of the polymer layer, polymer-coated CNDs have a longer circulation lifetime in the bloodstream, are less vulnerable to immune detection, and aggregate more in target locations. Moreover, polymer coatings may help encapsulate a wide range of drugs, enabling CNDs to operate as dual or multi-drug delivery systems [223].

### 5.9. Polymer Nanocomposites: Innovation and Future Directions

The nanocomposites are still being widely researched, particularly in fields that benefit from their excellent mechanical, thermal, and electrical properties. Polymer nanocomposites are advancing, with a focus on improved material design, process optimization, and broadening their applications beyond traditional uses.

#### 5.9.1. Self-Healing and Responsive Polymer Nanocomposites

Recent advances in polymer nanocomposites have led in the development of self-healing materials that can repair themselves. Composites like these can restore structural integrity after damage by integrating nanoparticles that trigger polymerization when a hole occurs or inserting microcapsules with healing agents. Self-healing nanocomposites are ideal for use in high-stress environments such as aerospace, where durability is critical [224]. Responsive polymer nanocomposites are designed to change their properties in reaction to environmental factors such as temperature, pH, and mechanical stress. These materials are helpful for applications that need adaptive properties, such as drug delivery systems that release therapeutic substances based on disease-specific conditions [225].

#### 5.9.2. Bio-Based and Environmentally Friendly Nanocomposites

Bio-based nanocomposites made from renewable polymers and biodegradable materials are gaining popularity as a result of environmental measurements. Polymers derived from maize starch, cellulose, and polylactic acid are used to create eco-friendly nanocomposites that disintegrate at the end of their lifetimes, reducing environmental impact [226]. The use of naturally occurring nanofillers, such as cellulose nanocrystals and lignin nanoparticles, enhances the mechanical and thermal properties of bio-based composites. These materials have promise in packaging, pharmaceutical, and consumer product applications, where environmentally friendly materials are becoming increasingly popular [227].

#### 5.9.3. Advanced Nanofiller Integration Techniques

Traditional techniques of incorporating nanofillers into polymer matrices frequently have problems in attaining uniform dispersion, which is critical for good composite characteristics. To overcome this issue, innovative technologies like electrospinning, shear mixing, and in situ polymerization have been developed to improve nanofiller distribution and integration. These approaches enable the production of nanosized reinforcing networks, resulting in composites with higher qualities when compared to those produced using traditional methods. This improved dispersion is critical in high-performance applications like flexible electronics and electromagnetic interference shielding [228].

### 5.10. Polymeric Carbon Nanodots for Sensor Technologies and Environmental

Polymeric CNDs have established themselves as useful components in sensor technology due to their fluorescence, conductivity, and ability to be selectively functionalized. CND-based sensors are versatile enough to detect a wide spectrum of chemical and biological analytes with good sensitivity and selectivity [229].

#### 5.10.1. Fluorescence-Based Sensors

The photoluminescent characteristics of CNDs are critical to their use in fluorescence-based sensors. By altering their surface with analyte-specific recognition elements, CNDs can detect changes in fluorescence intensity, color, or wavelength in the presence of target molecules. For example, CNDs functionalized to detect heavy metals or poisons exhibit an apparent shift in fluorescence upon binding to the target, allowing for real-time monitoring of environmental contaminants [230].

#### 5.10.2. Electrochemical Sensing Applications

CND electrochemical sensors are good for detecting biomolecules such as glucose or neurotransmitters due to their high conductivity and huge surface area. Surface functionalization with specialized enzyme immobilization, such as glucose oxidase for glucose detection, permits CND-based sensors to attain high sensitivity while maintaining low detection limits, which is critical for point-of-care diagnostics and environmental monitoring [231].

### 5.11. Polymeric Carbon Nanodots in Nanomedicine

Polymeric CNDs are increasingly used in nanomedicine due to their low toxicity, excellent biocompatibility, and adjustable characteristics. Photothermal treatment (PTT) is one area of active research in which CNDs absorb near-infrared (NIR) light and convert it to heat, therefore targeting cancer cells while minimizing damage to surrounding healthy tissues. Surface-functionalized CNDs may be adjusted to specific wavelengths, increasing their penetration and therapeutic efficacy [232]. When paired with chemotherapy (chemothermal treatment), CNDs enhance the cancer-killing effects, a method that is showing promise in early tests. Another promising area is gene and nucleic acid delivery. CNDs can be functionalized to bind siRNA or DNA, boosting cellular intake and minimizing genetic material destruction by circulatory enzymes. This feature solves a critical difficulty in gene therapy: delivering genetic material accurately to target cells while avoiding an immune reaction [233]. Furthermore, dual imaging and drug delivery applications are expanding, with CNDs acting as both a contrast agent for imaging and a carrier for drug administration. These multifunctional CNDs provide real-time tracking of medication distribution in the body, thus enhancing therapy efficacy and providing personalized treatment monitoring [234].

### 5.12. Structural Innovations in Polymer Nanocomposites

Developments in polymer nanocomposites include not just the incorporation of nanofillers, but also the engineering of internal structures to improve strength, flexibility, and functional capabilities. For example, layer-by-layer assembly processes allow for fine control over composite thickness and nanofiller arrangement, resulting in materials with unique mechanical characteristics, such as strong impact resistance or controlled elasticity. Core–shell nanocomposites, in which a core material is wrapped in a protective or functional shell, are being developed for application in harsh settings, such as aerospace. These composites can withstand high temperatures and prevent radiation deterioration, which is critical for materials used in satellite and spacecraft components. In addition, 3D printing technologies enable the development of complicated, customized nanocomposite structures. Researchers may change the qualities of 3D-printed materials for smart watches, flexible sensors, and sophisticated prostheses by changing the polymer matrix and filler content [235]. This versatility marks a huge step forward in material design, enabling for the exact customization of nanocomposites on demand.

**Figure 6:**
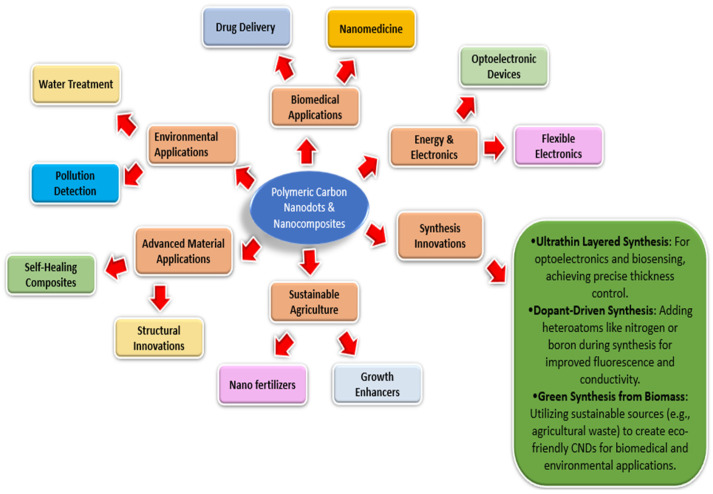
Various applications of polymeric carbon nanocomposites.

Both polymeric CNDs and polymer nanocomposites have potential in sustainable agriculture and environmental applications. CNDs as Nano fertilizers provide a unique approach to precise nutrient delivery. Plants might take in vital nutrients more efficiently after loading them onto CNDs, decreasing fertilizer waste and lowering environmental impact. Some research suggests that CNDs can boost plant growth and stress tolerance, allowing crops to tolerate drought, pests, and other environmental stresses. In water treatment, polymeric CNDs are being developed as photocatalysts to break down organic contaminants and remove heavy metals [236]. CNDs’ large surface area and adaptable reactivity make them perfect for breaking down pollutants when exposed to sunlight, which is both efficient and ecologically benign. In addition, membrane-based water purification systems that use polymer nanocomposites show tremendous potential for filtering out tiny pollutants, since the embedded nanofillers increase both filtration performance and mechanical durability [237]. Various applications of polymeric carbon nanocomposites are demonstrated in Figure 6.

## 6. Applications for Carbon Nanodots in Drug Delivery

### 6.1. Overview of Drug Delivery Systems

**Figure 7:**
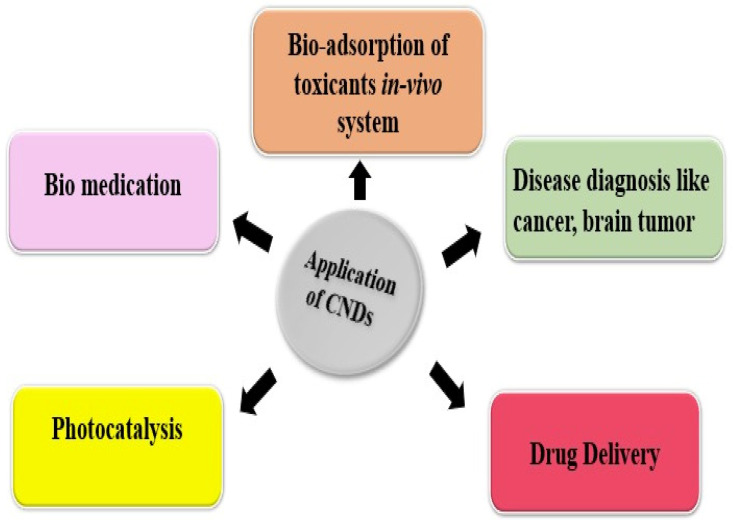
Different applications of carbon nanodots in biomedical domain.

Drug delivery systems, or DDS, are essential to the progress of medicine because they aim to improve the convenience, safety, and effectiveness of therapeutic interventions. These systems entail the planning and execution of methods for releasing and transferring medications in a regulated way inside the body [238]. Their main objectives are to enhance medication pharmacokinetics, boost bioavailability, and guarantee targeted distribution to particular tissues or cells. Drug delivery systems comprise an array of methodologies, ranging from conventional oral tablets to cutting-edge nanotechnology-based carriers. This thorough analysis looks at the different kinds, intrinsic difficulties, and possible advancements in medication delivery systems in the future. In the past, oral pills, capsules, injections, and topical creams were the main traditional forms used for drug administration. These techniques had drawbacks despite their efficacy, such as unstable release profiles, low bioavailability, and poor drug solubility [239]. The development of sophisticated medication delivery systems has been prompted by the demand for more accurate and patient-friendly methods. There are several kinds of drug delivery systems. In order to increase patient compliance, oral drug administration options include both traditional tablets and capsules and controlled-release formulations. Rapid onset of action is ensured by injectable techniques like intramuscular and intravenous injections. Topical medication delivery offers localized therapies through the use of lotions, gels, ointments, and transdermal patches. Metered-dose inhalers and dry powder inhalers are two methods of inhalation medication administration that specifically target the respiratory system [240]. Liposomes, polymeric nanoparticles, and dendrimers are examples of nanoparticle-based drug delivery agents that provide targeted and controlled release. While implantable drug delivery systems, including drug-eluting implants, progressively release medications for localized treatment, microneedle-based systems enable transdermal drug delivery [241]. Approaches like antibody–drug conjugates (ADCs) and nanoparticle targeting, which improve specificity, are included in targeted medication delivery. Drug delivery systems have difficulties even with their importance. For drug carriers to be safely integrated into biological systems, biocompatibility and toxicity reduction are essential. For a treatment to be effective, it must maintain drug stability, achieve appropriate drug loading, and regulate release kinetics [242]. There are obstacles in overcoming biological barriers, like the blood–brain barrier, and immunogenicity is still an issue. Reproducibility and cost-effectiveness concerns must be addressed in order to scale up production for commercial use. Drug delivery has a bright future ahead of it with many developments and opportunities. Personalized medicine could lead to the creation of medication delivery methods specific to a person’s genetic composition. Sensitive materials and sensor integration enable smart drug delivery systems to adjust to changes in the body’s physiology. It is hoped that advanced nanocarriers would enable advances in gene and RNA therapy [243]. Emerging horizons include bioelectronics and digital medication delivery systems that may monitor patient responses and modify drug release accordingly. Furthermore, 3D-printing technology has the potential to completely transform the way drugs are delivered by enabling the development of personalized systems with exact control over composition and structure. Drug delivery systems have experienced tremendous development and now provide a variety of methods to improve therapeutic interventions. Drug delivery technologies are changing the face of medicine, from traditional techniques to cutting-edge nanotechnology. The future will be characterized by overcoming obstacles and adopting cutting-edge technologies, with an emphasis on smart systems, personalized medicine, and cutting-edge treatments [244]. Below, Figure 7 demonstrates the various applications of carbon nanodots.

### 6.2. Role of Carbon Nanodots in Enhancing Drug Delivery

**Figure 8:**
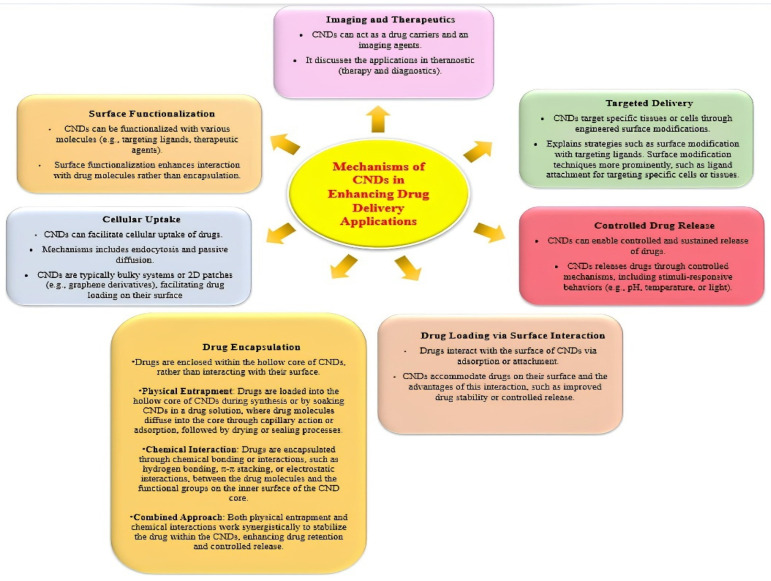
Role of CNDs in drug delivery enhancement.

Utilizing the special qualities of these nanoscale carbon-based materials, carbon nanodots (CNDs) can be used to improve the delivery of drugs, opening up new possibilities in nanomedicine and addressing issues related to traditional drug administration. CNDs are interesting candidates for drug delivery applications because of their unique properties, which include biocompatibility, fluorescence, and a high surface area [245]. The capacity of CNDs to increase the solubility and bioavailability of medications is one of their main advantages. Drug research frequently encounters the challenge of poor solubility, which restricts the potency of medicines [246]. Because of their large surface area, CNDs can be used as carriers for hydrophobic medications, improving their solubility and allowing enhanced body absorption. Furthermore, one essential quality that guarantees low toxicity and compatibility with biological systems is the biocompatibility of CNDs. Drug carriers must possess this attribute since any negative effects might compromise the treatment’s effectiveness and safety. Because of their biocompatibility, CNDs can be used in a variety of drug delivery methods, offering an effective way to transfer therapeutic substances with a low chance of side effects [247]. Drug delivery systems are being revolutionized by the optical features of CNDs, especially their intense fluorescence, which helps with real-time imaging and tracking. Because CNDs glow, drug carriers can be seen, making it possible for researchers to track their movements throughout the body and evaluate how well drugs are delivered. This has great potential for therapeutic applications, as well as research and development, providing a way to optimize and customize drug administration based on patient reactions [248]. The function of CNDs in drug distribution is further enhanced by surface functionalization. Targeting ligands, medicinal compounds, or other biomolecules can be attached to CNDs by simply adding different functional groups on their surface. Because of their adaptability, medication carriers can be made to match the way they interact with particular cells or tissues [249]. Drug release can be regulated and targeted by engineering functionalized CNDs to react to external stimuli, such as pH changes or the presence of particular enzymes. This degree of accuracy in medication administration has a key role in reducing side effects and raising the therapeutic index of medications. Because CNDs are tiny, interactions at the cellular and molecular levels are facilitated, which is beneficial for drug delivery. They can cross physiological barriers, such as the blood–brain barrier, which is a difficult therapy issue for neurological illnesses; their nanoscale size makes it possible. The delivery of medications to the central nervous system by CNDs shows promise in treating diseases like brain tumors and neurodegenerative disorders [250]. Beyond conventional small-molecule medications, CNDs show promise in providing a broad spectrum of therapeutic substances. They are appropriate transporters for proteins, peptides, and nucleic acids (such as DNA and RNA) due to their flexibility. This skill is especially important in the emerging field of gene and RNA therapies, because successful interventions depend on the targeted delivery of genetic material. The potential of CNDs in developing nucleic acid therapies and biologics is highlighted by their capacity to distribute and protect sensitive biomolecules. Another domain in which CNDs excel is the recently developing subject of targeted medication delivery. The surface of CNDs can be functionalized with certain ligands to enable the construction of drug carriers that bind to target cells or tissues with preference [223]. By using a targeted strategy, medication distribution becomes more particular, and therapeutic agents are delivered to their intended location more precisely. For example, CNDs can be designed to precisely target cancer cells during cancer treatment, reducing harm to healthy tissues and enhancing the overall therapeutic result. The special combination of characteristics that carbon nanodots possess, i.e., biocompatibility, fluorescence, surface functionalization abilities, and nanoscale dimensions, defines their role in improving drug delivery. Their ability to tackle issues related to traditional drug administration is facilitated by these characteristics. Carbon nanodots have the potential to completely transform drug delivery systems as this field of study develops, providing cutting-edge approaches for the precise, targeted, and controlled distribution of therapeutic substances in a variety of medical applications [251]. Figure 8 demonstrates the role of CNDs in drug delivery enhancement.

### 6.3. Mechanisms of Drug Delivery Using Carbon Nanodots

Since the introduction of nanotechnology, the field of drug delivery has advanced significantly. Of the various kinds of nanomaterials, carbon nanodots (CNDs) have attracted the most attention. On the nanoscale level, these carbon-based materials have special properties that make them attractive choices for enhancing drug delivery systems. In this extensive work, we explore the intricate mechanisms underlying CND-assisted drug delivery, examining how they can improve solubility, biocompatibility, surface functionalization, real-time imaging, and targeted drug administration [252].

#### 6.3.1. Increasing Bioavailability and Solubility

CNDs solve the issue of many medicinal chemicals’ poor solubility. Their ability to function as carriers for hydrophobic medicines stems from their enormous surface area and distinct surface chemistry. CNDs’ hydrophilic surface interacts well with water, making it possible for stable bonds to form with hydrophobic medicines and increases their solubility. Because of its increased solubility, the medication has a higher bioavailability, which increases the amount of the medicine that enters the bloodstream and enhances its therapeutic effect [253].

#### 6.3.2. Decreased Toxicity and Biocompatibility

Any successful drug delivery method must be biocompatible. As CNDs have low immunogenicity and cytotoxicity and are intrinsically biocompatible, they can be used in biological systems. They are a safe and well-tolerated substrate for drug administration because of their compatibility with biological surroundings, thus lowering the possibility of side effects. For CND-based drug delivery systems to be successfully integrated into clinical applications, this quality is necessary [254].

#### 6.3.3. Imaging and Tracking in Real Time

CNDs’ bright fluorescence is used to track and image drug distribution in real time. This optical characteristic makes it possible to monitor drug carriers inside the body non-invasively, giving important information on how they are distributed, accumulate, and are eliminated. In order to optimize drug delivery systems, real-time imaging is essential since it allows researchers to correctly monitor the movement of drug carriers and make sure they reach their intended targets. This capability holds promise for precise medication administration depending on the needs of each individual patient and tailored treatments [255].

#### 6.3.4. Functionalization of Surfaces

Surface functionalization expands CNDs’ potential for drug delivery. Through surface modification, CNDs can be designed to bind particular drug molecules, targeted ligands, or other biomolecules. This adaptation minimizes undesirable outcomes and improves drug-delivery precision by enabling selective targeting of specific cells or regions. Furthermore, it is possible to modify functionalized CNDs such that they react to outside stimuli like pH variations or the presence of specific enzymes, enabling precise drug release at the intended site. This flexibility is a valuable asset for accomplishing precise and efficient medication administration [256].

#### 6.3.5. Nanoscale Aspects for Improved Cellular Communication

CNDs can interact with cellular and molecular targets in an efficient manner due to their nanoscale size. Due to their tiny size, CNDs are able to pass across physiological barriers such as cell membranes and the blood–brain barrier (BBB). Drug transport to the central nervous system is significantly hampered by the BBB in particular. CNDs are useful for treating neurological illnesses because of their capacity to pass through these barriers and their adjustable surfaces, which open up novel possibilities for drug delivery to the brain. Their nanoscale characteristics improve the effectiveness of medication delivery and cellular communication [58].

#### 6.3.6. Transport of Different Therapeutic Agents

Delivering sensitive biomolecules while maintaining their functioning and integrity is made possible by CNDs, which offer a robust and biocompatible framework. Because of their versatility, they are useful instruments for developing biologics and nucleic acid therapeutics [257].

#### 6.3.7. Mechanisms for Targeted Drug Delivery

CNDs’ advanced surface functionalization methods allow for targeted medication delivery. CNDs can selectively bind to target cells or tissues by binding particular ligands to their surfaces, improving the accuracy of drug delivery. With CNDs specifically tailored to target cancer cells, this targeted strategy enhances therapeutic efficacy and decreases off-target effects, especially in cancer treatment. By raising the therapeutic index of drugs and reducing overall exposure, targeted drug delivery enhances patient outcomes [258].

## 7. Biomedical Applications for CNDS

CNDs have been widely used for various biomedical applications due to their excellent PL properties; wavelength-tunable emission properties; and unique electronic, mechanical, thermal, and chemical properties. In contrast to the traditional quantum dots (QDs), which essentially contain heavy metals, are known to show toxicity, and are environmentally hazardous, CNDs are safer and more non-toxic on both the cell and animal level, making them more environmentally friendly and biologically compatible for biomedical applications such as biosensing, bioimaging, and gene and drug delivery [259,260]. In this context, in the recent past, various kinds of sensors have been designed using CNDs to detect different targets, such as DNA [214], heavy metals [261], glucose [262], proteins [263], H_2_O_2_ [264], nitrite [265], phosphate [266], etc. For instance, CNDs prepared from flour and electrochemical carbonization of sodium citrate and urea show the PL emission which could be selectively quenched by Hg^2+^ with a detection limit of 0.5 nM and 3.3 nM, respectively [127,257]. Other heavy metals, such as Sn^2+^, Fe^3+^, Pb^2+^, Cr^6+^, Mn^2+^, and Cu^2+^ [261], were also detected using CNDs. The water-soluble CQDs prepared from lemon peel were used to design a highly sensitive fluorescent probe for the detection of Cr^6+^ ions with a detection limit of about 73 nM. Additionally, these CQDs were immobilized over electrospun TiO_2_ nanofibers. The photocatalytic activity for CQDs/TiO_2_ composite was seen to be about 2.5 times higher than pure TiO_2_ nanofibers when methylene blue dye was used as a model pollutant [126]. The CNDs modified with boronic acid have been utilized for nonenzymatic blood glucose sensing. This novel sensor could detect the glucose level in the range of 9-900 µM with a detection limit of 1.5 µM. The plasma glucose concentration determined by this method was observed to be in good agreement with the values measured by a commercial blood glucose monitor, thus proving the high efficiency of the produced CND-based sensor [267]. Recently, a CND-based fluorescence turn-on sensor was fabricated for hydrogen peroxide (H_2_O_2_) detection in aqueous solutions through a photo-induced electron transfer mechanism. The developed sensor exhibited good selectivity and sensitivity with a detection limit of 84 nM [268]. Table 1 presents examples of the application of CNDs in biosensors.

The application of CNDs as fluorescent labels for cellular imaging was first reported by Sun et al. CNDs are a promising candidate for bioimaging purposes because of their low side effects and toxicity, excellent water solubility, and visible-to-near-infrared (NIR) emission properties [141,263,269,270,271]. Jiang et al. [272] have reported the presence of photoluminescent (PL) CNDs in commercial Nescafe instant coffee with a size of 4.4 nm and QY of about 5.5%. Coffee-derived CNDs are directly applied in the imaging of carcinoma cells and small guppy fish without functionalization. CNDs are also passivated with PPEI-EI for two-photon luminescence microscopy, which is used to image human breast cancer MCF-7 cells [76]. CNDs exhibited bright PL both on the cytoplasm and in the cell membrane after a 2 h incubation at 37 °C. The cellular uptake of CNDs was temperature-dependent, with no internationalization observed at 4 °C. Similarly, Zhu et al. [273] prepared a two-photon “turn-on” fluorescent probe, where CNDs were used for imaging hydrogen sulfide in live cells and tissues.

Carbon dots are also proven to possess an excellent gene/drug-loading capability [274]. Besides their biocompatibility, nontoxicity, and photoluminescence properties, their small size and large surface area allow for rapid cellular uptake [275,276,277]. Recently, Lee et al. [274] demonstrated DOX delivery in vitro and in vivo using CNDs. The DOX was loaded on CNDs via electrostatic interactions with 95% loading efficiency and induced death of HepG2 and MCF-7 cancer cells, as well as tumors, in mice. Interestingly, pure CNDs preferably labeled the nucleus, whereas CNDs loaded with DOX were mainly distributed in the cytoplasm. Meanwhile, a CND coated alginate-based smart stimuli-responsive drug delivery system was proposed by Majumdar et al. [275]. Here, carbon dots were coated on the surface of alginate beads, and garlic extract (GE), which contains allicin, was taken as a model drug system. Interestingly, the amount of GE loaded on alginate beads coated with CNDs was 60% higher than uncoated alginate beads. The loaded system shows pH-dependent controlled drug release, which results in increased therapeutic efficiency and controlled drug release in relation to the amount of pathogen (MRSA) present in the target. Jiao et al. [276] developed a smart carrier for a redox-responsive controlled drug delivery system by grafting carboxyl-abundant CNDs to the surface of silica NPs, where drug release was seen to be highly pH-dependent. Here, DOX loaded onto the grafted silica have high drug loading, up to 13.1%, and exhibited a high cellular uptake and an excellent therapeutic effect on cancer cells by MTT (3-(4,5-Dimethylthiazol-2-yl)-2,5-diphenyl tetrazolium bromide) assay. Other than drug delivery, gene delivery has been also successfully demonstrated with positively charged CNDs by Yang et al. [277]. The CNDs with a positive charge link to plasmid DNA easily and efficiently transfect the therapeutic plasmid into cells with low cytotoxicity. Recently, Zhang et al. [278] demonstrated that hyaluronate (HA) and PEI-functionalized CNDs were internalized readily into the cytoplasm of cancer cells via HA-receptor-mediated endocytosis. These functionalized CNDs were found to have excellent gene condensation compatibility via electrostatic attraction and protective capacity by preventing nuclease degradation. Table 1 presents various examples of biomedical applications of CNDs.

**Table 1:** Examples of sources, preparation techniques, and biomedical applications of CNDs.

Sources of Carbon	Preparation Techniques	Size (nm)	Quantum Yield (%)	Color	Excitation Wavelength (nm)	Application	Ref.
Rose-heart radish	Hydrothermal	1.2–6.0	13.6	Blue	330	Sensing Fe^+3^	[279]
Prunus persica (peach)	Hydrothermal	8	15	Blue	325	Cellular imaging and oxygen reduction reaction	[280]
Trapa bispinosa peel	Hydrothermal	5–10	0.1	Green	365	Cellular imaging	[281]
Saccharum officinarum juice	Hydrothermal	3	5.67	Blue	365	Cellular imaging of bacteria and yeast	[282]
Unripe fruit extract of Prunus mume	Hydrothermal	9	16	Blue	355	Cellular imaging	[283]
Apple juice	Hydrothermal	4.5	4.27	Blue	360	Imaging of mycobacterium and fungal cells	[86]
Chionanthus retusus fruit extract	Hydrothermal	5	9	Blue	365	Metal ion sensing and imaging of fungal cells	[284]
Pseudo-stem of banana	Hydrothermal	1–3	48	Green	-	Sensing Fe^+3^, Imaging of Hela and MCF-7 cells *	[285]
Honey	Solvothermal	2	19.18	Blue	365	Sensing Fe^+3^ and imaging of Hep-2 and Hela cells *	[286]
Garlic	Hydrothermal	11	17.5	Blue	365	Cellular imaging and free radical scavenging	[287]
Sweet potato	Hydrothermal	3.39	8.64	Blue	365	Fe^+3^ sensing and cellular imaging	[288]
Walnut shell	Hydrothermal	3.4	-	Green	360–460	Cellular imaging	[289]
Glycerin and PEG	Microwave	3–4		Blue	365	Nitrite sensing	[290]
Bloomed algae	Microwave	8	13	Blue	365	In Vitro imaging	[291]
Tissue paper	Microwave	4.2	93	Blue	-	Determination of Glutathione	[292]
Kidney beans	Hydrothermal	20–30	8	green	340	Cellular imaging	[293]
Water Chestnut and onion	Hydrothermal	3.5	12	Green-Blue	400–600	Sensing of Cu (II) and Imaging of Coenzyme A	[294]
Food waste-derived	Ultrasonic	4.6	2.85	Blue-Red	400–470	In vitro bioimaging	[295]
Beer	Gel filtration chromatography	2.5	7.39	Blue	360	Breast cancer cell imaging and drug delivery	[296]
Lignin biomass	Ultrasonic and hydrothermal	2–6	21	Blue Green Red	310–420–540	Cellular imaging	[297]
Onion waste	Hydrothermal	15	28	Blue Green Red	408–488–561	Sensoring of Fe^3+^ and cellular imaging	[298]
Bee pollens	Hydrothermal	1–2	6–12.8	Blue-green	365	Cellular imaging and catalysis	[299]
Coriander leaves	Hydrothermal	2.4	6.48	green	320	Sensoring of Fe^3+^ and cellular imaging	[300]
Grape seed	Microwave	1–8	31.79	multicolor	250–550	Nucleus imaging and pH sensing	[301]
Carrot	Hydrothermal	2.3	7.60	Blue	365	Drug delivery	[287]
Sugarcane molasses	Hydrothermal	1.9	5.8	Blue	365	Sensoring of Fe^3+^ and cellular imaging	[302]
Mango leaves	Microwave	2–8	-	Red	325	Cellular imaging and temperature sensors	[303]
Papaya juice	Hydrothermal	3	7	Blue Green Red	365 488 561	Cellular imaging	[304]
Latex	Microwave	2–8	-	green	360–520	Metal sensing and cellular imaging	[305]
Mangosteen pulp	Hydrothermal	5	-	Blue	330	Sensoring of Fe^3+^ and cellular imaging	[306]
Lotus root	Microwave	9.41	19	Blue	360	Heavy metal ion detection and cellular imaging	[307]
Date kernel	Hydrothermal	2.5	12.5	Blue	365	Sensing of drugs and cellular imaging	[308]
Winter melon	Hydrothermal	4.5–5.2	7.51	Blue	360	Cellular imaging	[309]

* Hela, human epithelial carcinoma; HepG2, human hepatocarcinoma cells; MCF-7, breast cancer cell.

## 8. Challenges and Future Perspectives

### 8.1. Current Challenges in Carbon Nanodot-Based Drug Delivery

Carbon nanodots (CNDs) have emerged as promising candidates for drug delivery applications due to their unique physicochemical properties, biocompatibility, and potential for targeted therapy. However, several challenges persist in harnessing the full potential of CNDs for effective drug delivery systems. Understanding and addressing these challenges are critical for advancing this field towards practical biomedical applications.

One of the primary challenges in utilizing CNDs for drug delivery is the lack of standardized synthesis methods and characterization techniques. CNDs can be synthesized using various approaches, such as laser ablation, chemical oxidation, or microwave-assisted methods, leading to variations in size, surface chemistry, and optical properties. This diversity makes it difficult to establish universal protocols for drug loading, stability assessment, and performance evaluation [310]. Stability of CNDs under physiological conditions is crucial for their successful application in drug delivery. Many CNDs are prone to aggregation, which can compromise their dispersibility and effectiveness. Moreover, ensuring long-term biocompatibility and minimizing potential toxicity are ongoing challenges. The effects of CNDs on cellular function, metabolism, and immunogenicity require thorough investigation to ensure their safe use in vivo [311]. To improve CNDs’ biocompatibility, researchers have concentrated on improving their surface functionalization and production techniques. Potential harmful effects are lessened by the selection of precursor materials and the use of biocompatible polymers. By adding hydrophilic functional groups to the surface, including hydroxyl or amino groups, CNDs’ water solubility and biocompatibility are increased and negative interactions in biological systems are reduced.

Achieving high drug-loading capacity while maintaining controlled-release kinetics is another hurdle. As aforementioned, the surface chemistry of CNDs plays a pivotal role in drug loading efficiency and release behavior. Engineering CNDs with specific functional groups can enhance drug binding affinity and regulate release profiles. However, optimizing these parameters to achieve therapeutic concentrations at target sites remains a complex task. Enhancing targeted delivery and tissue-specific accumulation of drug-loaded CNDs is essential for maximizing therapeutic efficacy and minimizing off-target effects.

Scaling up the synthesis of CNDs for industrial production without compromising quality and cost-effectiveness is a practical challenge. Large-scale production methods must be developed to meet the demand for clinical translation. Additionally, cost-effective strategies for functionalizing CNDs and loading drugs efficiently are essential for widespread adoption in healthcare settings. Lastly, navigating regulatory pathways and demonstrating the safety and efficacy of CND-based drug delivery systems are critical steps towards clinical translation. Establishing robust preclinical models that accurately mimic human physiology and disease states is imperative. Addressing these regulatory and translational hurdles requires interdisciplinary collaboration between researchers, clinicians, and regulatory authorities [312,313].

### 8.2. Future Directions and Emerging Trends

Thanks to new developments in nanotechnology and developing trends, there is exciting potential for the use of carbon nanodots (CNDs) in medication delivery in the future. Looking ahead, several future directions and emerging trends are shaping the trajectory of CND-based drug delivery. Future efforts will concentrate on designing multifunctional CND-based nanoplatforms capable of simultaneous drug delivery, imaging, and therapy. Integrating functionalities such as targeting ligands, stimuli-responsive elements, and imaging agents into CNDs will enable precise control over drug release kinetics and enhance therapeutic efficacy [311]. Theragnostic CND platforms combining therapeutic and diagnostic capabilities will gain prominence. These systems can enable real-time monitoring of drug delivery, therapeutic response, and disease progression, thereby guiding personalized treatment strategies.

Smart CNDs provide focused and customized drug delivery, reducing adverse effects and maximizing therapeutic benefits. They do this by reacting to particular cues within the body, such as pH changes or the presence of biomolecules. Further advancements in surface engineering techniques will facilitate the development of CNDs with tailored physicochemical properties. Fine-tuning surface chemistry and structure can optimize biocompatibility, cellular uptake, and biodistribution, leading to improved pharmacokinetics and reduced off-target effects [314]. It is anticipated that the investigation of new biocompatible precursors and environmentally friendly synthesis techniques would solve environmental issues and improve the sustainability of CND-based drugs. Furthermore, research endeavors can concentrate on refining large-scale production techniques to expedite the transition of CNDs from laboratory-based to real-world clinical applications. Personalized drug delivery tactics based on unique patient characteristics may become possible as our understanding of the interactions between CNDs and biological systems expands. Customizing CNDs to a patient’s unique traits could transform treatment modalities by increasing effectiveness and reducing side effects. Fundamentally, the convergence of innovation, sustainability, and personalized medicine holds the key to the future of CND-based medication delivery, offering revolutionary approaches to a variety of health issues. Collaborative endeavors between academia, industry, and regulatory agencies will be essential for bringing CND-based drug delivery technologies to the bedside [315].

### 8.3. Patents and Publications on CNDs

Scientific literatures on CNDs based studies (Table 2). 

Scientific literature presented in tabular form on Patents published in CNDs study (Table 3).

## 9. Conclusions

The utilization of carbon nanodots (CNDs) as carriers for phytomedicine delivery holds significant promise in overcoming the limitations associated with traditional drug delivery systems. By leveraging the unique properties of CNDs, such as their tunable surface chemistry; high loading capacity on the surface and using hollow CNDs and nanosized dots, and biocompatibility, researchers can enhance the solubility, stability, bioavailability, and biocompatibility of phytomedicinal compounds. When considered as a drug delivery armament for phytopharmaceuticals, CNDs offer a wide range of applications which can be coupled with its high precision owing to its surface modifications. Its size being in the nanometer range allows for ease of penetration of various physiological barriers, something that is traditionally a challenge for phytopharmaceuticals. This property of CNDs is particularly advantageous in the treatment of cancers and neurological disorders crossing BBB and interacting via potential charge against target tissues with a polymeric nanocomposite combination. The review has highlighted various strategies for loading phytomedicine onto CNDs, including encapsulation and surface modification, as well as mechanisms for controlled release. This helps in targeting ligands for delivering the phytopharmaceuticals, leading to an increase in efficacy, as well as imaging with enhanced resolutions, even imaging of processes going on inside a living cell. This review emphasizes the various methods of synthesis of CNDs from different starting materials, their advantages, and few limitations, and it moves on to further discuss the use of such CNDs in theragnostic applications, especially as a drug delivery system for phytopharmaceuticals. CNDs could solve some major traditional problems associated with phytopharmaceuticals, i.e., poor stability, low solubility, and most importantly, low bioavailability coupled with poor pharmacokinetics parameters. However, challenges such as biocompatibility and toxicity concerns need to be addressed through rigorous research and development efforts. Looking ahead, continued advancements in CND-based drug delivery systems are expected to revolutionize the field of phytomedicine, paving the way for novel therapeutic interventions with enhanced efficacy and reduced side effects. CNDs possessing large surface areas may be able to interact with target marker biomolecules for impressive remediation against the desirable health issues. Moreover, large surface area-based phytopharmaceutical-mediated CNDs can be used as potential therapeutics for treating inflammation, gut microbial disorders, diarrhea, and neurodegenerations owing to their bio-adsorbent, cleansing, and toxin-diffusing bioremediation properties. However, the toxicity, functionalization, yields, and scalability of most CNDs need to be optimized and evaluated for significant biomedical application with impressive remedy against chronic health disorders in clinical models.

## Figures and Tables

**Table 2:** Publications of CNDs.

No.	Title	Findings	Results	Discussions	Ref.
1	“Effect of carbon nano-dots (CNDs) on structural and optical properties of PMMA polymer composite”	Strong intermolecular contacts, an improved amorphous phase, well-dispersed CNDs, increased photoluminescence, a shifted refractive index, and well-defined electron transitions were all displayed by the PMMA/CNDs nanocomposite films that were created using the solution cast process.	Amorphous PMMA/CNDs nanocomposite films with enhanced complexation and optical characteristics were created in this work. Improved photoluminescence and UV-VIS absorption point to the material’s applicability for photonic devices, LEDs, and other optoelectronic applications.	The stability, optical qualities, and amorphous phase of PMMA were all enhanced by the addition of CNDs. Their promise in nanotechnology devices is highlighted by their enhanced photoluminescence and UV-VIS absorption, which imply suitable for optoelectronic applications including LEDs and photodetectors.	[316]
2	“One-step synthesis and characterization of N-doped carbon nanodots for sensing in organic media”	The N-doped CNDs have useful applications in organic media without further functionalization due to their high quantum yield, excitation wavelength-dependent emission, and upconversion characteristics.	N-doped CNDs with 78% QY were created from PMA and showed excellent selectivity when it came to detecting nitroaromatic explosives by the quenching of fluorescence in organic environments.	In spite of their remarkable selectivity and possible commercial uses, such as self-cleaning surfaces, bioimaging of hydrophobic structures, and antiwetting, these N-doped CNDs hold great promise for the detection of nitroaromatic explosives.	[317]
3	“pH-dependent synthesis of novel structure-controllable polymer carbon NanoDots with high acidophilic luminescence and super carbon dots assembly for white-light-emitting diodes:	Different nanodot structures are produced by pH-dependent synthesis. White light from SCNDs is appropriate for LEDs. Investigation by PL reveals distinct emission paths. For carbon nanodots, theoretical computations reveal information on their electrical properties.	Changing the pH led to different topologies of carbon nanodots. Super-small carbon nanodots (SCNDs) showed promise for LED technology at pH < 1. They radiated white light. Unique emission channels were found via PL research, which improved knowledge of carbon-based fluorescence.	pH regulation provides customized nanodot morphology, which is essential for a variety of uses. The white emission of SCNDs offers opportunities for affordable LEDs. Understanding of carbon-based fluorescence is advanced by insights into PL processes, which direct future study.	[318]
4	“Freestanding luminescent films of nitrogen-rich carbon nanodots toward large-scale phosphor-based white-light-emitting devices”	Under UV light, CNDs produced vivid visible light that was appropriate for phosphor applications. Solid-state quenching was avoided via large-scale freestanding luminous films distributed across a polymer matrix, allowing for flexible, scalable, and thermally robust solid-state lighting systems.	Nitrogen-rich carbon nanodots (CNDs) with a restricted size range and a well-developed graphitic structure were produced by carbonizing polyacrylamide using an emulsion template. A high quantum yield of 40% was achieved in the fabrication of large-scale luminous films	CNDs with desired features were produced via synthesis using an emulsion-templated carbonization process. Because polymer matrix dispersion avoided quenching, flexible lighting systems on a vast scale could be made possible. Under realistic circumstances, white LEDs showed steady emission spectra, demonstrating the promise of CND-based solid-state lighting.	[319]
5	“Photoluminescence of argan-waste-derived carbon nanodots embedded in polymer matrices”	Excitation-dependent emission was demonstrated by blue-emitting CND-polymer nanocomposites, with the blue spectrum exhibiting the highest emission. By placing CNDs within optically transparent matrices, PLQY was increased by two to three times, reaching a 29.6% improvement.	For use in photonic conversion layers on solar PV cells, luminescent carbon nanodots (CNDs) derived from argan waste were distributed in poly(styrene-co-acrylonitrile) and cyclo-olefin copolymer matrices to generate thin films with a 30% PL conversion efficiency.	After being distributed in transparent polymers, CNDs made from argan waste maintained their long-term luminescence characteristics and increased radiative efficiency by two to three times. Because thin films are easily processed, they may be used as photonic down-conversion layers to improve the efficiency of solar cells, especially when UV light is used.	[320]
6	“Oxidative synthesis of highly fluorescent boron/nitrogen-co-doped carbon nanodots enabling detection of photosensitizer and carcinogenic dye”	P-CNDs with PEI passivation showed increased fluorescence after being synthesized in a simple manner. Protoporphyrin (PPD) introduction enabled fluorescence switch-off, allowing dye-doped nanoprobes with a limit of detection (LOD) of 9.9 pM−0.37 nM for Sudan red III (SRIII) and 15 pM for PPD.	The synthesis of boric acid and N-(4-hydroxyphenyl) glycine by hydrothermal oxidative method resulted in the straightforward production of carbon nanodots (CNDs) co-doped with silicon and nitrogen. polyethyleneimine (PEI) surface passivation improved fluorescence and monodispersity, resulting in polymerized CNDs (P-CNDs) with a 23.71% quantum yield.	Highly fluorescent B/N co-doped CNDs are easily synthesized and have potential uses in a number of fields. The surface passivation of PEI enhances monodispersity and fluorescence. PPD and SRIII may be detected with high sensitivity using dye-doped nanoprobes, indicating the possibility of useful sensing and detection applications.	[321]
7	“Fluorescent nitrogen-doped carbon nanodots synthesized through a hydrothermal method with different isomers”	N-oxide group production was aided by the o-PD precursor, whereas “lattice N” functionalities were inserted by hydrothermal synthesis using the m-PD precursor. While strongly quenched in propylene glycol methyl ether acetate (PGMEA), fluorescence was bright in polar solvents. Radiative emission from N-atom substitutions and N- and O-rich edge groups produced an ultrahigh quantum yield.	Using o-, m-, and p-phenylenediamine (PD) isomers, N-functionalized carbon nanodots (CNDs) were created hydrothermally, allowing for exact control of the N/C atomic ratio (20.2–25.7 at.%). In polar solvents, CNDs showed up to 99% ultrahigh quantum yield of strong fluorescence.	N-functionalized CNDs can have their characteristics precisely tuned by the hydrothermal process, which has applications in optical, sensing, energy storage/conversion, and biological devices. Strong fluorescence in polar solvents suggests that high-performing nanomaterials may find use in a range of fields.	[322]
8	“Synthesis of carbon nanodots from sugarcane syrup, and their incorporation into a hydrogel-based composite to fabricate innovative fluorescent microstructure polymer optical fibres”	The synthesis of CNDs is scalable, economical, and sustainable. Because of their N and O-rich edge groups, functionalized CNDs showed excellent quantum yields (85–99%), which increased their potential for use in optical and sensing applications.	Using a home microwave oven, CNDs with a 3 nm diameter and low polydispersity were created from sugarcane syrup. They were added to an optical fiber and fluorescent hydrogel composite and displayed fluorescence.	This work offers a green synthesis approach for CNDs that might potentially replace costly and harmful compounds used in optical fibers. The novel hydrothermal method improves the fluorescence and application of CNDs by precisely controlling N-functionalization.	[323]
9	“Synthesis of highly stable red-emissive carbon polymer dots by modulated polymerization: from the mechanism to application in intracellular pH imaging”†	This study offers a green technique for the synthesis of CNDs that might replace costly, hazardous compounds. R-CPDs’ surface state and crosslink enhanced emission (CEE) effect are the sources of their red emission. In optical fibers, they exhibit excellent stability, biocompatibility, and appropriateness for intracellular pH monitoring in HeLa cells. The novel hydrothermal method improves the fluorescence and application of CNDs by precisely controlling N-functionalization.	Red-emissive carbon polymer dots (R-CPDs) were synthesized at 80 °C and shown resistance to photobleaching, stability in high salinity, high pH sensitivity (pH 4–6), and adjustable solvent-color effect (λem 528–600 nm).	This study presents a straightforward, controlled technique for producing highly stable, biocompatible long-wavelength emitting R-CPDs. On the processes of photoluminescence, cellular uptake, and multifunctional uses, more study is required.	[324]
10	“Synthesis of surface molecularly imprinted poly-o-phenylene diamine/TiO_2_/ carbon nanodots with a highly enhanced selective photocatalytic degradation of pendimethalin herbicide under visible light”	Adsorption and selectivity were enhanced by the imprinted cavities and particular recognition sites on MIP. Photocatalytic activity was increased by the redshifted absorption to visible areas and the lowered band gap energy. The primary species responsible for PM photodegradation were O_2_% radicals.	Using PM herbicide as a template, a TiO_2_/CNDs/MIP nanocomposite was created. Under visible light, it demonstrated a high adsorption capacity (86.1 mg/g), good selectivity, and increased photodegradation efficiency (95%).	The TiO_2_/CNDs/MIP nanocomposite, with high stability and reusability, effectively adsorbs and degrades PM due to its unique structure. It offers a promising photocatalyst for environmental pollutant removal, leveraging lower energy to produce reactive species.	[325]
11	“Direct solvent-derived polymer-coated nitrogen-doped carbon nanodots with high water solubility for targeted fluorescence imaging of glioma”	N-CNDs demonstrated superior dispersibility, showed minimal cytotoxicity, and improved passive targeting to facilitate glioma fluorescence imaging. NMP was used in the synthesis as a source of carbon and nitrogen, as well as a solvent.	A direct solvothermal process was used to create pN-CNDs, which produced 5–15 nm particles with a quantum yield of 8.4%, sustained fluorescence, and great water solubility. They facilitated in vivo fluorescence imaging by penetrating glioma cells.	An effective method for producing functional carbon nanomaterials with promise for glioma-targeted imaging is the straightforward solvothermal synthesis of pN-CNDs. Their chemical makeup, growth, and targeting methods require more investigation.	[326]
12	“Evolution and synthesis of carbon dots: from carbon dots to carbonized polymer dots”	Different from conventional CNDs, CPDs are characterized by partial carbonization of polymer clusters. The lack of control over structure and performance in current synthesis methods limits their applicability in biolabeling, sensing, LEDs, and other areas.	The unique polymer/carbon hybrid structure of CPDs, a novel type of carbon dots, is apparent. Different bottom-up synthesis techniques show how synthesis circumstances affect the structures and characteristics of CPDs.	Future studies should concentrate on comprehending the principles of CPD synthesis, reaction mechanisms, and formation processes in order to achieve regulated synthesis and maximize their potential for use in a variety of disciplines.	[188]
13	“Design, synthesis, and functionalization strategies of tailored carbon nanodots”	Tuning the emissive, electrochemical, and chiroptical characteristics of the CNDs was made possible by regulating the reaction conditions. Their surface chemistry was further modified by post-functionalization, which increased their potential for use in a variety of applications, including energy conversion, sensing, and imaging.	They synthesized nitrogen-doped carbon nanodots (CNDs) that generate blue light by a bottom-up, microwave-assisted hydrothermal process. These CNDs were effectively utilized in hybrid and composite systems and showed tunable optoelectronic characteristics.	CNDs are appropriate for biomedical and energy applications due to their low cost, low toxicity, and strong photostability. Controlling synthesis for improved structural and performance regulation should be the main goal of future study, since this will increase their usefulness in many other domains.	[327]
14	“Facile synthesis of multicolour photoluminescent polymer carbon dots with surface-state energy gap-controlled emission”	The multicolor photoluminescence of PCNDs was found to be mostly controlled by the surface state, namely the C=N functional groups. As the C=N concentration rose, the band gap shrunk, and the emission peak moved.	Hydroquinone and ethylenediamine were used to develop multicolor emissive PCNDs that emitted green, blue, and yellow fluorescence. These PCNDs demonstrated outstanding solubility, high stability, and wavelength-independent photoluminescence upon stimulation.	A brand-new, gentle, and simple process was created to create PCNDs with outstanding water solubility and brilliant, steady emissions. A thorough characterization revealed how important surface states are in dictating the photoluminescence characteristics of PCNDs.	[328]
15	“Synthesis, separation, and characterization of small and highly fluorescent nitrogen-doped carbon nanodots”	NCNDs demonstrated strong luminescence, excellent fluorescence quantum yields (up to 0.46), and ease of functionalization. The surface states, which are impacted by various emission centers and traps, have a significant impact on the fluorescence.	Using a microwave-assisted process, nitrogen-doped CNDs (NCNDs) were created, producing particles with a narrow size distribution, adjustable fluorescence emission, and superior water solubility. Their size and surface characteristics were further improved using size-exclusion chromatography.	NCNDs with exceptional optical characteristics were manufactured via a straightforward, programmable microwave-assisted approach that controlled both surface and size. These adaptable NCNDs may find use in biomedicine, bioimaging, and optoelectronics	[329]
16	“Ultrahigh-yield synthesis of N-doped carbon nanodots with down-regulating ROS in zebrafish”	With a larger C=C percentage, the synthesis yield of CNDs rose by 3.3 times. By adding nitrogen in the forms of pyridinic-like N (74%) and NH2 (26%), the antioxidative qualities against ROS were strengthened.	A novel approach using carbon-carbon double bonds achieved a record-breaking 85.9% yield in synthesizing nitrogen-doped carbon nanodots (CNDs). These CNDs significantly reduced reactive oxygen species (ROS) by 68% in zebrafish.	A viable method for creating antioxidative nitrogen-doped CNDs is the idea of increasing synthesis yield via carbon–carbon double bonds. These CNDs may be used as nanodrugs to treat illnesses associated with ageing.	[330]
17	“A tumor microenvironment responsive mesoporous polydopamine theragnostic probe embedded with Gd/I-doped carbon nanodots for CT/MR/FL imaging and chemo/photothermal synergistic therapy”	A probe using mesoporous polydopamine (PDA) and Gd/I-doped carbon nanodots was developed for targeted tumor imaging and combined chemo/photothermal therapy. This probe responded to tumor microenvironment.	This probe increased the imaging contrast, as well as the therapeutic efficacy both in vitro and in vivo. Significant inhibition in tumor was observed when both chemotherapy and photothermal modalities were combined.	This theragnostic probe is a promising strategy for tumor imaging and personalized therapy. This can easily overcome limitations of single-modality cancer therapy whilst minimizing side effects.	[331]
18	“Printable Thermo- and Photo-stable Poly(D,L-lactide)/Carbon Nanodots Nanocomposites via Heterophase Melt-Extrusion Transesterification”	They developed Poly (D, L-lactide) (PDLA)/ carbon nanodots which had enhanced thermal and photostability. This was achieved through heterophase melt-extrusion transesterification. This also enhanced the mechanical properties of the CNDs significantly.	The resulting CNDs had showed excellent stability under induced photo and thermal stress with high resistance to degradation. They also retained their optical properties.	Retention of optical properties with high mechanical strength indicates utility in sensors and packaging materials. Melt-extrusion transesterification also provides a simple, scalable method to produce high performance CNDs.	[332]
19	“Decagram-Scale Synthesis of Multicolor Carbon Nanodots: Self-Tracking Nanoheaters with Inherent and Selective Anticancer Properties”	This study was able to develop a multicolor CND in decagram quantities. These CNDs had selective anticancer effects and self-tracking properties.	CNDs functioned as both fluorescent agent with strong photothermal conversion efficiency and nanoheaters for photothermal therapy against cancer whilst minimal toxicity to healthy cells. In vivo tests also confirmed these.	This can be an economical and scalable method to produce multicolor CNDs with dual properties that can aid in diagnostics, as well as therapy.	[333]
20	“Hyaluronic acid dressing of hydrophobic carbon nanodots: A self-assembling strategy of hybrid nanocomposites with theragnostic potential”	Authors introduced a self-assembly strategy to create hybrid nanocomposites by dressing CNDs with Hyaluronic Acid. This strategy enhanced the biocompatibility and stability of CNDs along with their use as a theranostic agent.	HA-CNDs had excellent water dispersibility and biocompatibility. This also had excellent tumor targeting abilities due to the affinity of hyaluronic acid towards CD44 receptors of cancer cells. In vitro and in vivo tests demonstrated their capabilities of diagnostics, as well as drug delivery and therapy.	This hybrid system offered a simple multifunctional CND with theranostic applications especially in cancer diagnosis and treatment. This is a promising candidate for less invasive and personalized cancer diagnostics and therapy.	[334]
21	“Charge-Convertible Carbon Dots for Imaging-Guided Drug Delivery with Enhanced in Vivo Cancer Therapeutic Efficiency”	This study developed charge-convertible CDs which enabled controlled drug release leading to improved tumor targeting efficiency. These also had fluorescence and MRI capabilities.	These CDs showed excellent in vivo performance with increased accumulation in tumor tissues. pH responsive behavior of these CDs allowed efficient drug release at acidic tumor microenvironment.	This presents a novel strategy for image guided drug delivery in cancer therapy with improved therapeutic outcomes and minimal side effects. Ability to release drug in acidic tumor microenvironment further makes this approach, a chance at personalized cancer therapy with higher efficacy.	[335]
22	“NIR Emissive Carbon Nanodots as a Tool to Mark Ribosomal RNA and Components using Super-Resolution Microscopy”	This study developed near infrared (NIR) emissive CNDs for labelling components of a live cell especially rRNA. These CNDs showed stability, biocompatibility and NIR fluorescence for high-resolution imaging of cellular structures.	CNDs were able to label ribosomal components, ribosome distribution within the cell, as well as visualization of rRNA. This provided insights into ribosomes that were not detectable with traditional imaging.	This highlights the importance of NIR emission CNDs in studying live cellular components with high resolution, especially ribosomal function and assembly. This marks a new approach for investigating molecular mechanisms of various synthesis and processes within the cell.	[336]
23	“Controlled delivery of sildenafil by β-Cyclodextrin-decorated sulfur-doped carbon nanodots: a synergistic activation of ROS signaling in tumors”	This study developed a drug delivery system for controlled release of sildenafil to enhance reactive oxygen species production in tumor cells with the help of β-cyclodextrin decorated sulfur-doped CNDs. This is an approach to improve synergistic ROS signaling activation by sildenafil.	The sulfur-doped CNDs delivered sildenafil in a controlled manner which enhanced the cytotoxic effects of sildenafil in tumor cells due to increased oxidative damage. These demonstrated improved therapeutic outcomes in comparison to free sildenafil, in vivo.	This is a promising approach for controlled drug delivery and tumor specific therapy to enhance anticancer activity by using ROS pathway.	[337]

**Table 3:** Patents on CNDs.

No.	Title	Patent Number	Findings	Date	Ref.
1	“Metal-enhanced photoluminescence from carbon nanodots”	US10837904B2	The invention enhances the detectable emissions of carbon nanodots through Metal-Enhanced Fluorescence (MEF). By positioning carbon nanodots at an optimal distance from plasmon-supporting materials like silver island films, this technique significantly improves brightness, photostability, and detectability, making it highly effective for biological imaging applications.	17 November 2020	[338]
2	“Nanomaterials with enhanced drug delivery efficiency”	US11478433B2	Based on medicinal natural products, supramolecular particles improve bioavailability, increase stability in acidic conditions, and distribute therapeutic agents efficiently, leading to better treatment results for diabetes or tumors.	25 October 2022	[339]
3	“Carbon nanodot, and preparation method and application thereof”	CN102849722B	The technique solves quenching problems in the production of highly fluorescent carbon nanodots, which may be used for cryptography, photovoltaics, and biological imaging, among other things. It is straightforward and inexpensive.	29 August 2012	[340]
4	“Nanocarbon composite structure having ruthenium oxide trapped therein”	US7572542B2	The nanocarbon composite, incorporating ruthenium oxide within graphene via Ketjen black and ultracentrifugal reaction, exhibits enhanced electrochemical activity, making it suitable for high-capacity capacitor applications in electrical energy storage.	10 June 2005	[341]
5	“Traditional Chinese medicine bio-based carbon nanodots, preparation method thereof, fluorescent probe, traditional Chinese medicine pharmaceutical preparation and application”	CN111778018A	The innovation proposes a carbon nanodot based on ginsenoside that has a large number of surface functional groups that allow for flexible alterations and good stability. It is biocompatible with other substances and functions as a potential biological fluorescence probe. It also shows selective inhibition on PC12 cells.	8 June 2020	[342]
6	“Preparation and regulation method of high-colour quality fluorescent carbon nanodots”	CN109504375B	Using this technique, standard white-light emission and near-spectrum matching with sunlight are achieved, yielding high-quality fluorescent carbon nanodots with good color attributes.	12 December 2018	[343]
7	“Carbon nanodot compound and preparation method thereof, fluorescent powder and light source material”	CN106833631B	A stable carbon nanodot complex with a silicon dioxide covering is presented in this invention, providing improved fluorescence qualities and resilience to environmental influences.	4 February 2017	[344]
8	“Fused carbon dot, preparation method and application thereof”	CN113403068B	This innovation includes fused carbon dots manufactured by a straightforward, economical technique that exhibits improved near-infrared emission and good light–heat conversion.	16 June 2021	[345]

## List of Abbreviation

CND	Carbon nanodot
CNDs	Carbon nanodots
DNA	Deoxyribonucleic acid
RNA	Ribonucleic acid
BBB	Blood–brain barrier
EPR	Enhanced Permeability and Retention
EDTA	Ethylene Diamine Tetra Acetic acid
TEM	Transmission Electron Microscopy
GQDs	Graphene quantum dots
CNDs	Carbon dots
PDs	Polymer dots
CNPs	Carbon nanoparticles
QCE	Quantum confinement effect
PL	Photoluminescence
CD	Carbon dots
QY	Quantum yield
H_2_SO_4_	Sulfuric acid
HNO_3_	Nitric acid
NaNO_3_	Sodium nitrate
KmNO_4_	Potassium permanganate
NaOH	Sodium hydroxide
H_2_O_2_	Hydrogen peroxide
CQDs	Carbon Quantum Dots
TiO_2_	Titanium dioxide
MW	Microwave
NAC	N-acetyl-L-cysteine
PEG200	NH2-polyethylene-glycol
NPs	Nanoparticles
DDS	Drug delivery systems
DOX	Doxorubicin
ADCs	Antibody–drug conjugates
PNCs	Polymer nanocomposites
CTS	Chitosan
sADM	S-Adenosylmethionine
NDs	Nanodrugs
EPR	Enhanced Permeability and Retention
LEDs	Light-Emitting Diodes
PEG	Polyethylene glycol
PLA	Polylactic acid
PTT	Photothermal treatment
NIR	Near-infrared
EACs	Ehrlich ascites carcinoma cells
MTT	(3-(4,5-Dimethylthiazol-2-yl)-2,5-diphenyl tetrazolium bromide)
HA	Hyaluronate
PMMA	Polymethyl Methacrylate
NMP	N-Methyl-2-Pyrrolidone
N-CNDs	Nitrogen-Doped Carbon Nanodots
pN-CNDs	Phosphorus and Nitrogen-Doped Carbon Nanodots
CPD	Carbon polymer dots
SCNDs	Super-small carbon nanodots
UV	Ultraviolet
LOD	Limit of Detection
PEI	Polyethyleneimine
PPD	Protoporphyrin
PLQY	Photoluminescence Quantum Yield
P-CNDs	polymerized CNDs
SR III	Sudan red III
PGMEA	Propylene glycol methyl ether acetate
R-CPDs	Red-emissive carbon polymer dots
CEE	Crosslink enhanced emission
PCND	Photoluminescence CND
NCNDs	Nitrogen-doped CNDs
ROS	Reactive oxygen species
MEF	Metal-Enhanced Fluorescence
